# A comprehensive analysis of carotenoids metabolism in two red-fleshed mutants of Navel and Valencia sweet oranges (*Citrus sinensis*)

**DOI:** 10.3389/fpls.2022.1034204

**Published:** 2022-10-18

**Authors:** Jaime Zacarías-García, Paul J. Cronje, Gianfranco Diretto, Lorenzo Zacarías, María Jesús Rodrigo

**Affiliations:** ^1^ Departamento de Biotecnología de Alimentos, Instituto de Agroquímica y Tecnología de Alimentos (IATA), Consejo Superior de Investigaciones Científicas (CSIC), Valencia, Spain; ^2^ Citrus Research International (CRI), Department of Horticultural Sciences, University of Stellenbosch, Stellenbosch, South Africa; ^3^ Italian National Agency for New Technologies, Energy, and Sustainable Development (ENEA), Biotechnology Laboratory, Casaccia Research Center, Roma, Italy

**Keywords:** citrus fruit, red orange, carotenoids, lycopene, colourless carotenes, gene expression, abscisic acid

## Abstract

Kirkwood Navel and Ruby Valencia are two spontaneous bud mutations of the respective parental lines of sweet orange (*Citrus sinensis*) Palmer Navel and Olinda Valencia, showing an atypical red pigmentation of the pulp. These red-fleshed varieties are commercially available and highly attractive for consumers but their carotenoid metabolism and the basis of the mutation have not been investigated. The red colour of Kirkwood and Ruby pulp was observed from the very early stages of fruit development until full maturity and associated with an altered carotenoid profiling. The red-fleshed varieties accumulated from 6- up to 1000-times more total carotenoids compared to the standard oranges. Specifically, the pulp of Kirkwood and Ruby accumulated large amounts of phytoene and phytofluene, and moderate contents of lycopene. Moreover, the red-fleshed oranges contained other unusual carotenes as δ-carotene, and lower concentrations of downstream products such as β,β-xanthophylls, abscisic acid (ABA) and ABA-glucosyl ester. This peculiar profile was associated with chromoplasts with lycopene crystalloid structures and round vesicles likely containing colourless carotenes. The flavedo and leaves of Kirkwood and Ruby showed minor changes in carotenoids, mainly limited to higher levels of phytoene. The carotenoid composition in Kirkwood and Ruby fruits was not explained by differences in the transcriptional profile of 26 genes related to carotenoid metabolism, covering the main steps of biosynthesis, catabolism and other processes related to carotenoid accumulation. Moreover, sequence analysis of the lycopene cyclase genes revealed no alterations in those of the red-fleshed oranges compared to the genes of the standard varieties. A striking event observed in Kirkwood and Ruby trees was the reddish coloration of the inner side of the bark tissue, with larger amounts of phytoene, accumulation of lycopene and lower ABA content. These observation lead to the conclusion that the mutation is not only manifested in fruit, affecting other carotenogenic tissues of the mutant plants, but with different consequences in the carotenoid profile. Overall, the carotenoid composition in the red-fleshed mutants suggests a partial blockage of the lycopene β-cyclization in the carotenoid pathway, rendering a high accumulation of carotenes upstream lycopene and a reduced flow to downstream xanthophylls and ABA.

## Introduction

*Citrus* is one of the major fruit-tree crops in the world (Talón et al., 2020) with an estimated production of more than 140 million tons and is highly demanded worldwide for fresh consumption and juice processing (https://www.fao.org/faostat/en/#data/QCL). The health-related and sensorial properties of *Citrus* and the consumers perception of their benefits are closely associated with the content of certain phytochemicals such as vitamins, carotenoids, minerals, flavonoids, phenolic acids, limonoids, volatiles and sugars, among others (Ma et al., 2020).

Fruit colour is one of the most remarkable features among the different citrus species and varieties, and a key factor in quality and consumer acceptance (Tadeo et al., 2020). The colouration of most citrus fruits is due to carotenoids, a large family of isoprenoid-derived compounds, which play essential roles in many physiological processes of the plant (Rodriguez-Concepcion et al., 2018). The variability in carotenoid content and composition determines the distinctive pigmentation of citrus fruits, ranging from yellow or pale-yellow to orange and the pink/red of specific varieties of pummelos, grapefruits and sweet oranges (Xu et al., 2006; Ikoma et al., 2016; Liu et al., 2016; Lu et al., 2017; Tadeo et al., 2020).

Carotenoids are recognized for their benefits in human nutrition and health (Eggersdorfer and Wyss, 2018; Amengual, 2019; Ma et al., 2020). They are efficient antioxidants scavenging singlet molecular oxygen (^1^O_2_) and peroxyl radicals (Stahl and Sies, 2003; Müller et al., 2011) and specific carotenoids such as α-carotene, β-carotene and β-cryptoxanthin are the main source of vitamin A from plant-based food (Eggersdorfer and Wyss, 2018). In addition, many studies pointed out that the regular intake of carotenoids from fruit and vegetable products has important health-related benefits and reduces the risk of specific degenerative diseases (Rao and Rao, 2007; Rajendran et al., 2014; Böhm et al., 2021; Meléndez-Martínez et al., 2021). Consequently, the colouration of citrus fruits, determined by the content and composition of individual carotenoids, has a remarkable impact not only in their organoleptic quality but also regarding their nutritional and health properties.

**Figure 1:**
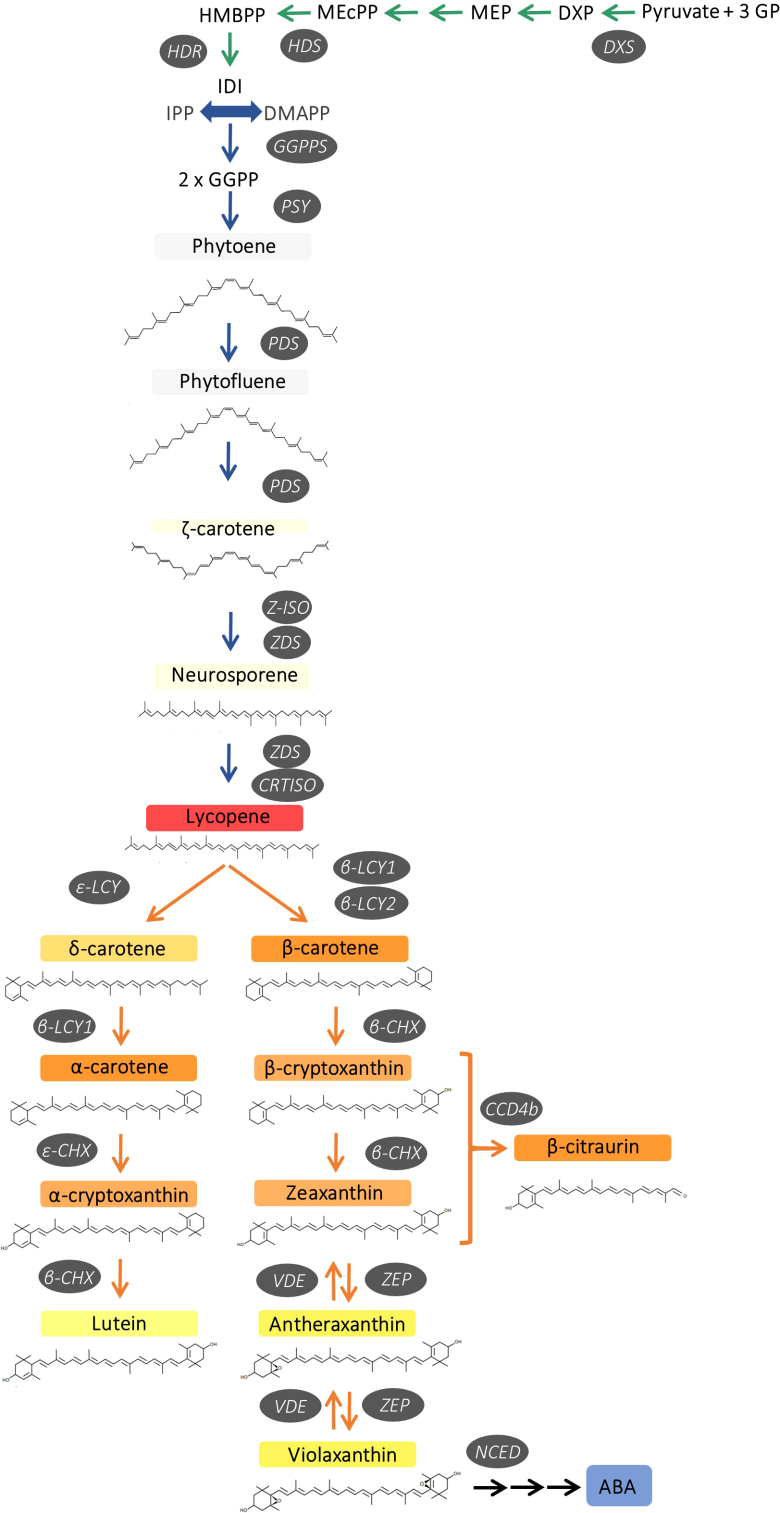
Schematic representation of carotenoid biosynthesis in citrus fruits, indicating main enzymes and genes of the pathway. 1-deoxy-D-xylulose-5-phosphate synthase (*DXS*), hydroxymethylbutenyl diphosphate synthase (*HDS*) and reductase *(HDR*), geranylgeranyl diphosphate synthase (*GGPPS*), phytoene synthase (*PSY*), phytoene desaturase (*PDS*), ζ-carotene isomerase (*Z-ISO*), ζ-carotene desaturase (*ZDS*), carotene isomerase (*CRTISO*), ϵ-lycopene cyclase (ϵ-LCY), β-lycopene cyclase (*β-LCY1/2*), ϵ-carotene hydroxylase (ϵ*-CHX*), β-carotene hydroxylase (*β-CHX*), carotenoid cleavage dioxygenase 4b (*CCD4b*), zeaxanthin epoxidase (*ZEP*), violaxanthin de-epoxidase (*VDE*) and 9-cis-epoxy-carotenoid dioxygenase (*NCED*).

The biosynthesis of carotenoids in plants occurs exclusively in plastids through the methylerythritol 4-phosphate (MEP) pathway, which provides precursors for carotenoid production. This pathway involves the participation of several enzymes, such as 1-deoxy-D-xylulose-5-phosphate synthase (DXS), which is the first reaction of the MEP pathway and catalyses the synthesis of 1-deoxy-d-xylulose 5-phosphate from pyruvate and D-glyceraldehyde 3-phosphate. Then, the terminal reactions of the MEP pathway are catalysed by 4-hydroxy-3-methylbut-2-enyl diphosphate synthase (HDS), which synthesize (E)-4-hydroxy-3-methyl-but-2-enyl diphosphate (HMBPP), and HMPP reductase (HDR), an enzyme that converts HMBPP into isopentenyl diphosphate (IPP) and dimethylallyl diphosphate (DMAPP). The condensation of one DMAPP (DMAP) and three IPP molecules by the geranyl geranyl pyrophosphate synthase (GGPS) produces geranylgeranyl diphosphate (GGPP) (**Figure 1**). The first committed step for carotenoid biosynthesis in plants is the head-to-head condensation of two GGPP molecules by phytoene synthase (PSY) to produce the first C40 carotenoid product 15-*Z*-phytoene. This reaction is generally considered the major rate-limiting step of carotenogenesis. Sequential desaturations and isomerizations by phytoene desaturase (PDS), ζ-carotene isomerase (Z-ISO), ζ-carotene desaturase (ZDS) and carotenoid isomerase (CRTISO) produce lycopene, through the intermediates ζ-carotene and neurosporene (Moise et al., 2014). At this point, the pathway splits into two branches by cyclization of lycopene at both ends to give α-carotene (β,ϵ-branch) and β-carotene (β,β-branch). The β,ϵ-branch requires both lycopene ε-cyclase (ϵ-LCY) and lycopene β-cyclase (β-LCY) activities for the formation of ϵ- and β-ionone rings, respectively, at both ends to generate α-carotene. In the ε,β-branch, α-carotene is di-hydroxylated at both ionone rings by carotene hydroxylases (β-CHX/CYP97A and ε-CHX/CYP97C) to render lutein, the predominant carotenoid pigment in the photosynthetic membranes of green plant tissues (Kato, 2012). In the β,β-branch, a single activity β-LCY is required to introduce β-ionone ring at both ends of lycopene to form β-carotene (**Figure 1**).

In citrus fruit, two different β-LCY enzymes catalyse the cyclization of lycopene to β-carotene (Alquézar et al., 2009; Zhang et al., 2012). *Β-LCY1* is expressed in a large variety of tissues showing a fairly constant expression during fruit ripening, while β-LCY2 is preferentially expressed in fruits and is up-regulated during ripening at the onset of colouration (Alquézar et al., 2009; Zhang et al., 2012). In some citrus species, including sweet orange, two alleles of *β-LCY2* (a and b) with different activity to convert lycopene into β-carotene have been identified (Alquézar et al., 2009; Zhang et al., 2012; Lu et al., 2016). Subsequently in the pathway, β-carotene is converted to zeaxanthin *via* β-cryptoxanthin by two-step hydroxylation catalyzed by the carotene β-ring hydroxylase (β-CHX). Furthermore, zeaxanthin is converted to violaxanthin *via* antheraxanthin by zeaxanthin epoxidase (ZEP) (**Figure 1**). The accumulation of carotenoids depends on a tight relationship between synthesis and catabolism. In this context, carotenoid cleavage dioxygenases (CCDs) are a group of enzymes that catalyse the oxidative cleavage of carotenoids and, in citrus fruits, NCEDs (9-Z-epoxycarotenoid dioxygenases), CCD1 and CCD4b have been studied in relation to the catabolism of carotenoids (Kato et al., 2006; Ma et al., 2013; Rodrigo et al., 2013a). Moreover, the esterification of β,β-xanthophylls with fatty acids by xanthophylls acyl esterases (XES/PYP) is an active process in orange-pigmented citrus fruits which may enhance their stability and accumulation (Mariutti and Mercadante, 2018; Lux et al., 2019; Zacarías-García, et al., 2021).

Carotenoid synthesis and colour development in citrus fruit occur concomitantly with the differentiation of chloroplast into chromoplasts (Lado et al., 2015; Lu et al., 2017; Zhang et al., 2019), leading to the development of sink structures organized to store carotenoids, and in which fibrillins (FIB) may play structural roles organizing carotenoids in lipoprotein complexes (Simkin et al., 2007). Globular chromoplasts are the most representative in mature sweet orange fruits, characterized by abundant plastoglobules containing the β,β-xanthophylls (Lado et al., 2015; Schweiggert and Carle, 2017). By contrast, in tissues of varieties and mutants that accumulate lycopene, other type of chromoplasts containing needle-like crystals are observed (Lado et al., 2015; Zeng et al., 2015; Zhang et al., 2019).

The peel of immature-green sweet orange shows the characteristic carotenoid profile of a chloroplast-containing tissue, being lutein the main carotenoid, while the content of carotenoids in the pulp is almost negligible. At the onset of fruit colouration, a progressive decrease in lutein takes place in parallel with an accumulation of specific β,β-xanthophylls, being 9-Z-violaxanthin the main carotenoid in tissues of mature sweet oranges (Ikoma et al., 2016; Lux et al., 2019; Tadeo et al., 2020). On the contrary, accumulation of lycopene in citrus fruits is an unusual feature only described in certain varieties of pummelo (*Citrus grandis, Citrus maxima*), grapefruit (*Citrus paradisi*), or mutants of lemon (*Citrus limon*) and sweet orange (*Citrus sinensis*). Currently, only three red-fleshed sweet orange varieties have been characterized: Shara (Monselise and Halvey, 1961), Cara Cara (Saunt, 2000) and Hong Anliu (Liu et al., 2007). Exhaustive research has focused on the pattern of carotenoid accumulation in these red-fleshed citrus fruits with the aim of elucidating the molecular mechanisms responsible for this trait. The profile of carotenoids suggests that the basis of the alteration leading to lycopene accumulation may differ among citrus species and varieties (Liu et al., 2007; Alquézar et al., 2008, Yu et al., 2012; Alquézar et al., 2013; Zeng et al., 2015; Lu et al., 2016; Yan et al., 2018; Lana et al., 2020; Promkaew et al., 2020). In the red Hong Anliu, a spontaneous mutant of the Anliu orange, the concentration of carotenoids in the juice sacs was 2-times higher than in the standard variety, mainly by the accumulation of lycopene (~2 μg g^-1^) at expenses of xanthophylls, which are strongly reduced (Liu et al., 2007). In contrast, carotenoid compliment in the Cara Cara orange, a bud mutation of Washington Navel, has a different biochemical phenotype characterized by: 1) accumulation of considerable amounts of phytoene from early stages of development, that may reach up to 25-80 μg g^-1^ FW in mature fruits, 2) lycopene content in the pulp can be as high as ~9 μg g^-1^ FW, and 3) xanthophyll levels lower or similar than in the standard Navel (Xu et al., 2006; Alquézar et al., 2008; Rodrigo et al., 2015; Lado et al., 2015; Lu et al., 2017; Liu et al., 2021).

Overall, the molecular basis of lycopene accumulation in *Citrus* is not fully understood. In the red Star Ruby grapefruit pulp, for instance, a reduced expression of the *β-LCY2* gene compared to the white Marsh grapefruit has been suggested as the main feature for the lycopene accumulation (Mendes et al., 2011; Alquézar et al., 2013). The comparison of several pummelos varieties with different pulp pigmentation indicated that lycopene is associated with a reduced expression of genes encoding for enzymes operating downstream lycopene (Liu et al., 2016; Yan et al., 2018; Promkaew et al., 2020). On the contrary, in the pulp of Pink lemon, a spontaneous bud mutant of Eureka lemon, accumulation of lycopene was associated with an up-regulation and down-regulation of the genes upstream and downstream lycopene β-cyclase, respectively (Lana et al., 2020). While in Cara Cara orange, an enhancement of the expression of specific MEP pathway genes and an increased channelling of isoprenoid precursors into the carotenoid pathway, has been hypothesized to be related to its red-fleshed phenotype (Alquézar et al., 2008; Lu et al., 2017; Zhang et al., 2021), even though other mechanisms may be involved (Alquézar et al., 2008; Ye et al., 2010; Lu et al., 2017). Finally, in the Hong Anliu mutant, an up-regulation of genes upstream of lycopene and the preferential expression of the afunctional allele *β-LCY2b*, are suggested to enhance the accumulation of lycopene (Xu et al., 2009; Lu et al., 2016).

The availability and comparison of new lycopene-accumulating mutants may be a very valuable tool to dissect and understand the biological basis of lycopene accumulation in the pulp of sweet orange. Recently, two independent bud mutations, referred to as ‘Kirkwood Navel’ (Kirkwood, K) and ‘Ruby Valencia’(Ruby, R), from the standard Palmer Navel in Kirkwood Eastern Cape and the Olinda Valencia in Crocodile Valley (Mpumalanga), respectively, were identified in South Africa and selected for their red pigmentation of the pulp of mature fruits (https://www.citrogold.co.za/) (Barry et al., 2020). These mutants have been further propagated and the red-fleshed phenotype remained stable under commercial conditions. The accessibility of these two mutants is of genetic interest since each belongs to two distinctive orange groups: Navel and Valencia oranges. Moreover, both mutants are relevant for the commercial citrus industry since Navel are the main oranges for fresh-fruit consumption, whereas Valencia cultivars are the major source for juice production worldwide (Barry et al., 2020). Thus, this work aimed to perform a comparative physiological, biochemical and molecular characterization of the carotenoid metabolism in the red-fleshed mutants Kirkwood Navel and Ruby Valencia orange during fruit development and maturation. We report a comprehensive analysis of carotenoid content and composition in the pulp of both mutants and the corresponding standard varieties from the initial stages of fruit development to full maturity. The carotenoid data were complemented with the analysis of the expression of genes related to carotenoid metabolism, covering the MEP pathway (*DXS*, *HDS*, *HDR* and *GGPS*), carotenes and xanthophylls biosynthesis (*PSY*, *PDS*, *Z-ISO, ZDS*, *ϵ-LCY*, *β-LCY1*, *β-LCY2*, *β-CHX*), carotenoids catabolism (*NCED, CCD1* and *CCD4b*), storage and accumulation (*HSP21* and *FIB*), and xanthophylls esterification (*PYPs*).

## Material and methods

### Plant material, fruit size, colour index and internal maturity

**Figure 2:**
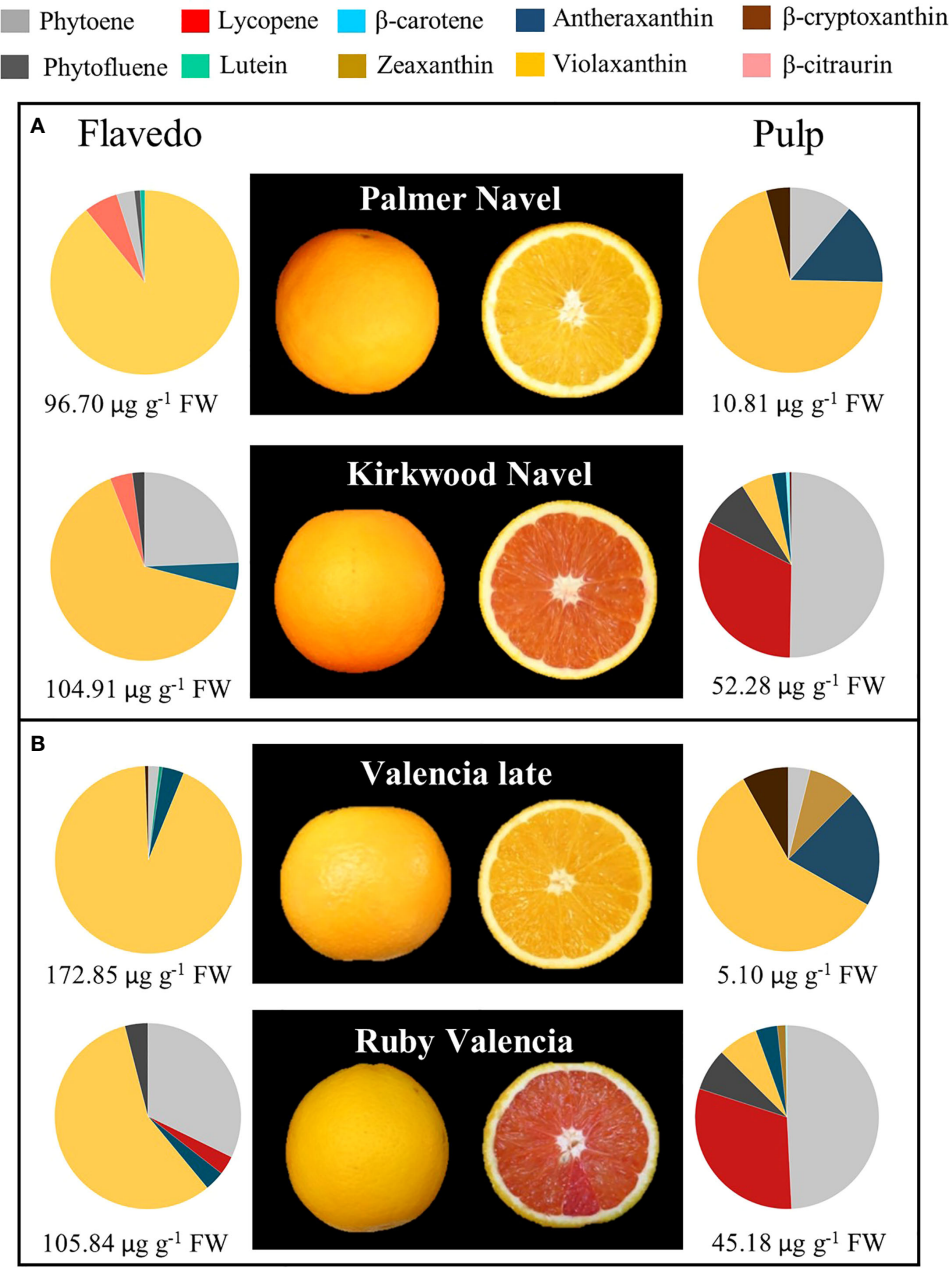
External and internal appearance and carotenoids content and composition of flavedo and pulp of mature fruits of Palmer Navel and Kirkwood Navel **(A)** and Valencia late and Ruby Valencia **(B)** oranges harvested in South Africa. The numbers below indicate total carotenoids concentration (µg g^-1^ FW).

Trees of the standard oranges (*Citrus sinensis* L. Osbeck) i.e., Foios Navel (referred as Navel, N) and Midknight Valencia (Valencia, V) as well as the two red-fleshed mutants Kirkwood Navel and Ruby Valencia were planted in the experimental orchard of the Foundation ANECOOP (Museros, Valencia, Spain; 39°34’10.8”N 0°21’28.8”W), growing in a loamy-sand soil with drip irrigation, and subjected to the same environmental and growing conditions (https://anecoop.com/en/leading-the-industry/new-varieties-and-new-products/the-doctors farm/). Adult trees of standard and mutant genotypes were located in continuous rows in the same evaluation block and both were budded onto Carrizo citrange [*Citrus sinensis* (L.) Osb. *× Poncirus trifoliata* (L.) Raf.] rootstock. Fruit of each genotype were periodically harvested starting at the initial stages of fruit development (June) until full maturation in January for Foios Navel and Kirkwood Navel, and April for Midknight Valencia and Ruby Valencia (**Figure 2**). For each sampling point, at least 20-30 fruit from three trees of each genotype were harvested and immediately delivered to the laboratory. Fruit were selected for uniform colour and size, and absence of any lesion or defect. Flavedo tissue (outer coloured peel layer of the fruit) was collected with a scalpel, and pulp juice vesicles were excised from central segments. Tissue was immediately frozen in liquid nitrogen, ground to a fine powder and stored at -80°C until analysis. Young-full developed leaves (3-4 months old) from the four genotypes were also collected from young shoots from the same trees. Leaves were rinsed with distilled water, dried before removing the central vein and freezing the leaves in liquid nitrogen before grounding it to a fine powder, and stored at -80°C.

Initial analysis of carotenoid content and composition were performed in mature fruits harvested from South Africa sampled from mature Palmer and Kirkwood Navel, and Valencia late and Ruby Valencia trees located in a cultivar evaluation orchard in Citrusdal (Western Cape, South Africa). All trees were budded onto Carrizo citrange [*Citrus sinensis* (L.) Osb. *× Poncirus trifoliata* (L.) Raf.] rootstock. The mature fruit of the Navel varieties were harvested in July and those of Valencia’s in October. The fruit were immediately delivered to the Department of Horticultural Sciences of the University of Stellenbosch (South Africa) and processed as stated above until analysis.

Branches of around 1 year-old (about 1 cm diameter) were removed from both mature Ruby Valencia and standard Valencia trees in the cultivar evaluation block in Citrusdal. At initial inspection, the Ruby Valencia shoots showed red colouration of the inner side of the bark. Sections around 5-10 cm long were excised and maintained in plastic bags to be transported to the University of Stellenbosch. Each section was cut into 0.5 cm long sections and the bark and xylem/wood tissues were separated with a scalpel, frozen in liquid nitrogen and lyophilized.

Colour of the peel and pulp was measured using a CR-400 Minolta chromameter (Konica Minolta, Japan) at three different positions on the fruit. Hunter parameters *a* (negative to positive corresponds from green to red) and *b* (negative to positive, from blue to yellow) were determined, and colour was expressed as the *a/b* Hunter ratio, a classical relationship for colour measurement in citrus fruits (Stewart and Wheaton, 1972). The data of fruit colour index for each developmental stage are the means ± SD of 10 fruits.

The juice was extracted from the pulp using a household electric hand squeezer (Citromatic MPZ22, Braun, Spain), filtered through a metal sieve with a pore size of 0.8 mm, and immediately frozen in liquid nitrogen and stored at -20°C until analysis. The total soluble solids (TSS) and total titratable acidity (TA) of the juice of each variety was determined using a digital refractometer PAL-BX/ACID1 (ATAGO, Japan). TSS was expressed as °Brix and TA as mg of citric acid/100 ml of juice. The maturity index (MI) was calculated as the TSS/TA ratio.

### Chlorophylls and carotenoids extraction

Chlorophylls and carotenoids were extracted and analysed as described by Rodrigo et al. (2015). Briefly, freeze-ground material of flavedo (0.5 g) and pulp (2 g), and lyophilized wood or bark (0.2 g) were weighed in screw-capped polypropylene tubes, 4 mL of methanol (MeOH) and 5 mL Tris-HCl (50 mM, pH 7.5) containing 1 M NaCl were added and each sample was sonicated 5 min in XUBA3 ultrasonic water bath (Grant Instruments, England) at room temperature. Dichloromethane (DCM) (10 mL) was added to the mixture, stirred for 5 min at 4°C and centrifuged at 3,000 g for 10 min at 4°C. The hypophase was recovered and the aqueous phase re-extracted with DCM until it was colourless. The extract was reduced to dryness by rotatory evaporator at 35°C and redissolved in diethyl ether to determine the chlorophyll (a+b) content by measuring the absorbance at 644 and 662nm (Smith and Benitez, 1955). Subsequently, samples were saponified in methanolic KOH (6%, w/v) overnight at room temperature. Saponified carotenoids were recovered from the upper phase after adding water and petroleum ether:diethyl ether (9:1) to the mixture. Extracts were dried and kept at −20°C until further analysis. Each sample was extracted at least twice, and results are mean ± SD.

### Carotenoids analysis by HPLC-DAD

The carotenoid composition of each sample was analysed by HPLC with a Waters liquid chromatography system equipped with a 600E pump and a photodiode array detector (DAD) model 2998, and Empower3 software (Waters, Spain). A C30 carotenoid column (250 × 4.6 mm, 5 μm) coupled to a C30 guard column (20 × 4.0 mm, 5 μm) (YMC, Teknokroma, Spain) was used. The chromatographic conditions are described in Lado et al. (2015) and Rodrigo et al. (2015). Absorbance spectra and retention time identified each carotenoid, peaks were integrated at their individual maximal wavelength, and their contents were calculated using the appropriate calibration curves, as described elsewhere (Lado et al., 2015; Rodrigo et al., 2015).

### Transmission electron microscopy

Sample fixation and preparation were carried out following the protocol described by Lado et al. (2020) with slight modifications. Briefly, juice vesicles were manually excised from freshly harvested fruit and fixed in modified Karnovsky fixative (3% glutaraldehyde solution K_2_PO_4_ 0.2 M, pH 7) followed by a post-fixation in OsO_4_ 2%. Subsequently, the tissue was dehydrated in ethanol and embedded in Spurr resin. Ultra-thin sections (0.1 mm) were cut and contrasted with a solution of uranyl acetate (4%) and lead citrate (0.5%). The samples were visualized on a JEOL JEM1010 transmission electron microscope (60 kV) (Microscopy Facility of the SCSIE-UV, Valencia, Spain).

### Gene expression analysis by quantitative real-time PCR

Total RNA extraction from flavedo and pulp was performed as described previously (Rodrigo et al., 2004; Alós et al., 2014). cDNA synthesis and gene expression analyses were performed as described by Rodrigo et al. (2013b), and subsequently treated with DNase treatment (Ambion, Madrid, Spain). Total RNA was quantified in a NanoDrop ND-1000 spectrophotometer (Thermo Fisher Scientific, Madrid, Spain). Briefly, 2 μg of total RNA were reverse transcribed using the SuperScript III Reverse Transcriptase (Invitrogen, Madrid, Spain) following the manufacturer’s instructions. Quantitative real-time polymerase chain reaction (qRT-PCR) was performed on a LightCycler 480 instrument (Roche, Madrid, Spain), using the LightCycler 480 SYBRGreen I Master kit (Roche, Madrid, Spain). Twenty ng of cDNA was used for each amplification reaction in a total volume of 10 μl. The cycling protocol consisted of 10 min at 95°C for pre-incubation, followed by 35 cycles of 10 s at 95°C for denaturation, 10 s at 59°C for annealing and 10 s at 72°C for extension. Fluorescence data were acquired at the end of extension phase and reactions specificity was checked by post-amplification dissociation curve. Amplification efficiency (E) and correlation coefficient (r^2^) of each primer were calculated using the standard curve method and the formula E = 10(−1/slope). For expression measurements, a LightCycler 480 Software release 1.5.0, version 1.5.0.39 (Roche, Madrid, Spain) was used, and the expression levels were relative to values of a reference sample calculated using the Software tool REST-MCS (Rest Multiple Condition Solver v2, http://rest.gene-quantification; Pfaffl et al., 2002). The selected reference sample to calculate expression levels was the standard Valencia pulp harvested in November, which was arbitrarily given the expression value of 1. The *Actin* gene expression was chosen to normalize raw Cp’s based on a previous selection of reference genes (Alós et al., 2014). The results were the average of three independent sample replicates and the relative expression Software tool REST 2009 (http://www.gene-quantification.de/rest-2009.html) was used to determine statistical significance between varieties (P < 0.05) The list of genes analysed and primers used for amplification are in **Table S4**.

### Sequencing of *ϵ-LCY*, *β-LCY1* and *β-LCY2* genes

Genomic DNA from pulp of immature fruits of Navel Foios, Valencia Midknight, Kirkwood Navel and Ruby Valencia was isolated using DNeasy Plant Mini Kit (Qiagen), and was used to amplify the genomic sequences of *β-LCY1* and *β-LCY2* from each genotype by PCR. Since genomic DNA of *ϵ-LCY* contains introns, cDNA from immature flavedo of the four varieties was used to amplify this gene. The primers used for amplification and sequencing were designed based on orange1.1g014377m.g for *β-LCY1*, orange1.1g010693m.g for *β-LCY2* (www.phytozome.com) and XM_025097735.1 for *ϵ-LCY* (www.ncbi.nlm.nih.gov). The full coding sequence of each gene and each variety was amplified using AccuPrime™Taq DNA Polymerase High Fidelity (Invitrogen, Massachusetts, USA). For *β-LCY1* and *β-LCY2*, the cycling program consisted of 30 s at 98°C, then 35 cycles of 10 s at 98°C for denaturation, 30 s at 58°C for annealing and 1 min at 72°C for extension. For *ϵ-LCY*, the cycling program consisted of 3 min at 98°C, 35 cycles of 30 s at 98°C for denaturation, 30 s at 56°C for annealing and 90 s at 72°C for extension. List of primers used for amplification and sequencing is supplied in **Table S4**. The single nucleotide polymorphisms in the sequences were analysed using Chromas 2.6.6. Software (Technelysium Pty Ltd; http://technelysium.com.au/), and the multiple sequence alignments were performed with DNAMAN sequence analysis software (Lynnon, Quebec, Canada).

### Analysis of abscisic acid and its catabolites

Determination of ABA, its glycosylated form (ABA-GE), and catabolites (phaseic and dihydrophaseic acid) in flavedo, pulp, bark and xylem of the different genotypes were performed as described by Diretto et al. (2020) with slight modifications. Briefly, frozen tissues were lyophilized and ground to a fine powder, and replicate samples of 200 mg of each genotype were extracted as previously described (Welsch et al., 2008). LC-HRMS was carried out using a Ultimate UHPLC-DAD (Dionex) coupled to a Q-Exactive quadrupole Orbitrap mass spectrometry System (Thermo Fisher Scientific), equipped with a C18 Luna column (150 x 2.0mm, 3μm) as described before (Noronha et al., 2022). Internal standard-based quantification was carried out using the MS data and the quantification software available in the Xcalibur 7.0 software package (Thermo Fisher Scientific, Bremen, Germany). Results are expressed as fold changes with respect to the internal standard.

### Statistical analysis

At least two biological replicates of each genotype were used for the different experiments of this study and the results are presented as the mean values ± standard deviation. Statistical differences between standard orange cultivars and the corresponding red-fleshed mutants were determined by an unpaired t-test, setting the significance level at p<0.05 by XLSTAT software.

## Results

### Characteristics of the red-fleshed Kirkwood Navel and Ruby Valencia oranges during fruit development and maturation

**Figure 3:**
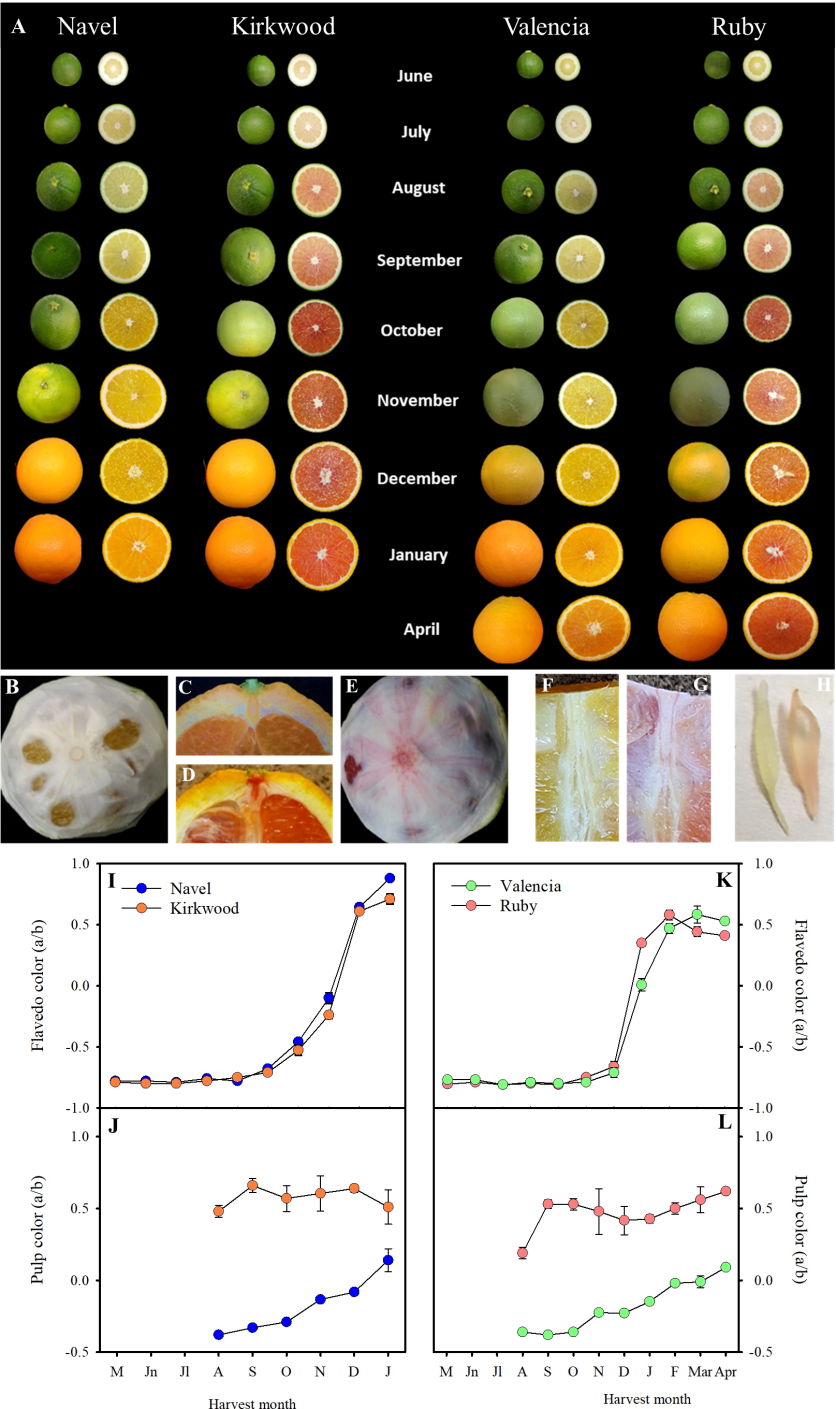
External and internal appearance of Navel, Kirkwood, Valencia and Ruby orange (*Citrus sinensis*) during fruit development and maturation, at the indicated harvest month. Fruits were harvested in the season 2018/2019, from an orchard located in Fundación ANECOOP (Museros, Valencia), Spain **(A)**. Mature fruits of Valencia **(B)** and Ruby **(E)** oranges, in which the external flavedo has been removed to see the color and the veins of the internal albedo layer. Longitudinal sections of the upper side of Valencia **(C)** and Ruby **(D)** oranges showing the notch of the peduncle and calix. Longitudinal section of the pulp of Valencia **(F)** and Ruby **(G)** oranges showing colour of the pulp and the central axis. Juice vesicle of Valencia (left) and Ruby (right) **(H)**. Evolution of the colour of the flavedo **(I, K)** and pulp **(J, L)** of Navel and Kirkwood, and Valencia and Ruby, respectively, during fruit development and maturation. Data are the means of 30 measurements ± SE.

Kirkwood Navel (K) and Ruby Valencia (R) are two bud independent mutations derived from the standard Palmer Navel and Olinda Valencia, respectively, distinguished by the red colouration of the flesh. However, fruits of K and V showed similar fruit morphology, external appearance, and colouration compared to their original lines (**Figures 2**, **3**).

As a first step for the physiological characterization of the altered colouration and biochemical compliment in both mutants, we performed an exploratory analysis of carotenoid composition in mature K and R fruits grown in a commercial orchard in the original country (Citrusdal, Western Cape, South Africa) in the season 2013/2014. The red-fleshed varieties showed an altered carotenoid profile both in the pulp and the flavedo (**Figure 2**). Total carotenoids in the pulp were nearly 5- and 9-times higher in K and R, respectively, than in the corresponding parental. The red pigmentation in the pulp of the mutants was associated with: 1) lycopene accumulation, at concentrations between 14 and 17 µg g^-1^ FW; 2) accumulation of high amounts of 15-*Z*-phytoene (>20 µg g^-1^ FW) and phytofluene (3-5 µg g^-1^ FW), and 3) reduced content of violaxanthin (up to 3 times lower in K than Palmer Navel). Despite the apparent normal orange colour of the flavedo in mutant fruits, total carotenoid content was similar in K and Palmer Navel but higher in Valencia (61%) than in R. Interestingly, the peel of both genotypes contained higher amounts of phytoene and reduced concentration of violaxanthin than the parental genotypes (**Figure 2**).

Further experiments were carried out on trees of K and R, and Foios Navel (N) and Midknight Valencia (V), as representatives of the standard parental varieties, grown in the same experimental orchard under Mediterranean environmental conditions of Valencia (Spain). N and K orange varieties are characterized by mid-season maturity and the period of harvesting would cover between December and February. Instead, Midknight Valencia and Ruby Valencia are late maturing oranges and generally harvested between April and May (Barry et al., 2020). Fruits were harvested from early stages of development to full maturity, covering eight developmental stages, from June to January in K and N, and nine stages, from June to April in R and V. The evolution of peel colouration, determined as the *a*/*b* Hunter ratio, were similar between fruits of the standard and the red-fleshed orange varieties (**Figures 3A, I, K** **)**. Mutant fruits did not display red pigmentation in the peel at any stage of maturation. On the contrary, differences in pulp colour between the standard and the red varieties were observed from the early stages of fruit development. From June onwards, the pulp of young fruitlets of both mutants showed a light-pink tint that turned more reddish as fruit developed (September), whereas fruits of N and V displayed greenish-yellow colour (**Figure 3A** **)**. From October to full maturation, K and R pulp developed a bright-red colouration (**Figures 3A, E, G** **)**, while N and V turned to the characteristic yellowish-orange tones of traditional oranges (**Figures 3A, B, F** **)**. The *a/b* Hunter ratio was higher in the pulp of K and R during the whole fruit development and maturation, exhibiting positive values since the early stages of fruit development (**Figures 3J, L** **)**. Moreover, the *a/b* Hunter ratio of K and R mature fruits was approximately 0.5, while N and V fruits displayed values around 0.1, in agreement with the colour exhibited by the corresponding juice vesicles (**Figure 3H**). It is worth to mention that other tissues of K and R, such as the calyx button and axial and central vascular bundle showed reddish pigmentation while the corresponding tissues of N and V fruits remained colourless or yellow-pigmented (**Figures 3B-G**).

Internal maturity index (MI) (°Brix/acidity) was determined at two ripening stages: at colour-break (October), and at full maturity, January for N and K, and April for V and R fruits. The MI of K was lower than in N at both ripening stages, while R showed similar levels to V (**Supplementary Tables 1, 2**). In general, internal maturation indexes in the red-fleshed mature fruits were virtually identical to the standard Navel and Valencia oranges.

### Carotenoids content and composition in standard Navel and Valencia, and the red-fleshed Kirkwood and Ruby oranges during fruit development and maturation

Carotenoid content and composition in the pulp and flavedo of K and R and the corresponding standard varieties were analysed during fruit development and maturation (**Tables 1**, **2** and **Supplementary Tables 5, 6**). Overall, the HPLC-DAD analysis identified twenty-six carotenoids in both fruit tissues (**Supplementary Table 3**).

**Table 1:** Carotenoid content and composition (µg g^-1^ FW) in the pulp of Navel and Kirkwood Navel at six different stages of development and maturation.

Carotenoids (µg g^-1^ FW)	Navel					
	June	July	September	October	December	January
Phytoene	3.20 ± 0.02*	traces	traces	traces	traces	nd
Phytofluene	0.16 ± 0.08*	nd	nd	nd	nd	nd
ζ-carotene	nd	nd	nd	nd	nd	nd
Neurosporene	nd	nd	nd	nd	nd	nd
Lycopene	nd	nd	nd	nd	nd	nd
δ-carotene	nd	nd	nd	nd	nd	nd
Lutein	0.17 ± 0.02	nd	0.18 ± 0.03	0.07 ± 0.01	0.38 ± 0.03*	0.66 ± 0.19*
β-carotene	nd	nd	nd	nd	nd	nd
A + Z	nd	nd	nd	0.04 ± 0.01	0.88 ± 0.12*	3.46 ± 0.11*
Violaxanthin	0.67 ± 0.09	nd	0.02 ± 0.01	0.77 ± 0.21	4.60 ± 0.22*	9.35 ± 0.83*
Other xanthophylls	0.22 ± 0.09	nd	nd	nd	1.04 ± 0.65	3.35 ± 0.30*
Total carotenoids	4.42 ± 0.42*	traces	0.20 ± 0.02*	0.99 ± 0.32*	6.91 ± 1.25*	16.82 ± 1.91*
**Carotenoids (µg g^-1^ FW)**	**Kirkwood**					
	**June**	**July**	**September**	**October**	**December**	**January**
Phytoene	37.55 ± 1.55	45.19 ± 2.15	53.8 ± 4.68	70.91 ± 3.30	79.20 ± 9.62	65.81 ± 4.48
Phytofluene	3.35 ± 0.13	7.8 ± 0.44	9.73 ± 1.14	9.02 ± 0.30	8.63 ± 0.50	14.97 ± 0.34
ζ-carotene	nd	nd	nd	nd	0.12 ± 0.02	0.45 ± 0.12
Neurosporene	0.12 ± 0.02	0.44 ± 0.07	1.01 ± 0.02	1.50 ± 0.16	nd	2.34 ± 0.03
Lycopene	0.19 ± 0.03	0.90 ± 0.09	2.09 ± 0.44	2.50 ± 0.20	5.25 ± 0.26	5.80 ± 0.06
δ-carotene	traces	0.20 ± 0.09	0.28 ± 0.02	0.60 ± 0.20	nd	1.01 ± 0.09
Lutein	nd	nd	nd	nd	0.27 ± 0.02	1.07 ± 0.14
β-carotene	0.15 ± 0.04	nd	0.15 ± 0.02	0.07 ± 0.01	0.34 ± 0.02	0.55 ± 0.21
A + Z	nd	nd	nd	0.08 ± 0.02	1.10 ± 0.03	2.82 ± 0.14
Violaxanthin	0.40 ± 0.15	nd	nd	0.72 ± 0.02	2.62 ± 0.01	3.12 ± 0.14
Other xanthophylls	0.07 ± 0.01	nd	nd	0.15 ± 0.06	0.46 ± 0.11	1.25 ± 0.66
Total carotenoids	41.83 ± 6.41	54.53 ± 8.57	67.06 ± 3.68	85.549 ± 0.99	98.08 ± 0.49	99.19 ± 6.86

A+Z is the sum of antheraxanthin and zeaxanthin. Violaxanthin is the sum of all *E*-violaxanthin, 9-*Z*-violaxanthin and luteoxanthin. Other xanthophylls are the sum of α-cryptoxanthin, β-cryptoxanthin, neoxanthin and non-identified xanthophylls (**Table S2**). Nd, no detected. Traces indicates concentrations lower than 0.05 μg g^-1^. Data are the mean of three biological replicates ± SD. Asterisks indicate significant differences between Navel and Kirkwood varieties by unpaired Student’s t-test (p<0.05).

**Table 2:** Carotenoid content and composition (µg g^-1^ FW) in the pulp of Valencia and Ruby at six different stages of fruit development and maturation.

Carotenoids (µg g^-1^ FW)	Valencia					
	June	July	September	October	January	April
Phytoene	traces	traces	0.03 ± 0.01*	traces	traces	nd
Phytofluene	nd	traces	0.15 ± 0.05*	nd	nd	nd
ζ-carotene	nd	nd	nd	nd	traces	0.09 ± 0.01*
Neurosporene	nd	nd	nd	nd	nd	nd
Lycopene	nd	nd	nd	nd	nd	nd
δ-carotene	nd	nd	nd	nd	nd	nd
Lutein	0.08 ± 0.01*	nd	nd	nd	nd	1.18 ± 0.47
β-carotene	nd	nd	nd	nd	nd	nd
A + Z	nd	nd	nd	0.09 ± 0.01	0.94 ± 0.04*	3.87 ± 0.05*
Violaxanthin	0.50 ± 0.03*	nd	nd	0.40 ± 0.01*	5.18 ± 0.01*	7.59 ± 0.10*
Other xanthophylls	0.18 ± 0.03	nd	nd	0.15 ± 0.17	0.98 ± 0.33	2.81 ± 0.78*
Total carotenoids	0.81 ± 0.18*	traces	0.18 ± 0.01*	0.69 ± 0.13*	7.10 ± 0.89*	15.45 ± 1.91*
**Carotenoids (µg g^-1^ FW)**	**Ruby**					
	**June**	**July**	**September**	**October**	**January**	**April**
Phytoene	17.76 ± 2.45	53.07 ± 3.58	65.7 ± 2.33	72.44 ± 0.59	87.15 ± 6.27	124.24 ± 0.95
Phytofluene	1.21 ± 0.21	4.6 ± 0.7	7.64 ± 1.14	10.94 ± 0.38	14.55 ± 0.37	24.48 ± 0.46
ζ-carotene	nd	nd	0.07 ± 0.03	nd	0.20 ± 0.01	1.24 ± 0.05
Neurosporene	nd	0.18 ± 0.02	0.53 ± 0.03	1.03 ± 0.14	0.63 ± 0.14	2.64 ± 0.01
Lycopene	traces	0.40 ± 0.02	0.17 ± 0.09	1.17 ± 0.21	2.90 ± 0.69	9.95 ± 3.05
δ-carotene	nd	0.05 ± 0.01	nd	nd	0.28 ± 0.09	1.05 ± 0.03
Lutein	0.31 ± 0.01	nd	0.15 ± 0.03	0.29 ± 0.012	0.32 ± 0.41	0.87 ± 0.57
β-carotene	nd	nd	nd	0.38 ± 0.01	0.24 ± 0.05	1.60 ± 0.01
A + Z	nd	nd	nd	0.09 ± 0.01	0.77 ± 0.05	3.52 ± 0.04
Violaxanthin	0.85 ± 0.01	nd	nd	0.12 ± 0.02	2.40 ± 0.22	4.10 ± 0.02
Other xanthophylls	0.18 ± 0.02	nd	nd	nd	0.35 ± 0.21	1.01 ± 0.19
Total carotenoids	20.36 ± 7.58	58.30 ± 3.51	74.26 ± 2.23	86.46 ± 0.21	109.79 ± 3.89	174.70 ± 5.34

A+Z is the sum of antheraxanthin and zeaxanthin. Violaxanthin is the sum of all E-violaxanthin, 9-Z-violaxanthin and luteoxanthin. Other xanthophylls are the sum of α-cryptoxanthin, β-cryptoxanthin, neoxanthin and non-identified xanthophylls (**Table S2**). Nd, no detected. Traces indicates concentrations lower than 0.05 μg g^-1^. Data are the mean of three biological replicates ± SD. Asterisks indicate significant differences between Valencia and Ruby varieties by unpaired Student’s t-test (p<0.05).

Differences in carotenoids content and composition in the pulp of the red-fleshed and standard oranges were evident from the early stages of development (June). Indeed, significant accumulation of phytoene and phytofluene occurred in K and R, accounting 90-99% of total carotenoids content, while N and V presented very little or negligible amounts of total carotenoids (**Tables 1**, **2**). The concentrations of phytoene and phytofluene substantially increased during ripening, and in the pulp of mature fruits of K and R reached levels as high as 80 and 148 µg g^-1^ FW, respectively, representing between 80-85% of the total content (**Tables 1**, **2**). Moreover, lycopene was detected at early stages in K and R, and its content increased from traces to 5.80 and 9.90 µg g^-1^ FW in mature fruits, respectively. Due to the significant accumulation of carotenes in the pulp of the red-fleshed varieties, the concentration of total carotenoids ranged from 6- to 1,000 times higher than the standard oranges (**Tables 1**, **2**). Other unusual carotenoids in citrus fruit, such as neurosporene, ζ-carotene and δ-carotene were also detected in variable proportions in the pulp of both mutants, while they were not detected in standard oranges (**Tables 1**, **2**). The concentration of these rare carotenoids in K and R pulps increased progressively during maturation until reaching contents of 1-2 µg g^-1^ FW in mature fruits. The presence of low or very low levels of β-carotene (<1 µg g^-1^) was quantified in mature pulp of K and R, while it was not detected in the standard oranges. Overall, the concentration of carotenes, including lycopene, in the pulp of both red-fleshed varieties increased steadily with ripening and the highest contents was reached at full maturity. The carotenoid profile of standard N and V oranges was characterized by the accumulation of β,β-xanthophylls since October, reaching the highest levels in January and April, respectively. The main carotenoid in mature fruits of both standard oranges was violaxanthin, followed by antheraxanthin and other β,β-xanthophylls. Even tough, β,β-xanthophylls also accumulated in K and R during maturation, their concentrations were between 2- and 3-times lower than in the standard oranges (**Tables 1**, **2**).

The flavedo of green fruits (September and October) of the four orange varieties showed the characteristic composition of chloroplastic tissues, being lutein, the predominant carotenoid followed by minor concentrations of β,β-xanthophylls (violaxanthin, neoxanthin, zeaxanthin and anteraxanthin) and α- and β-carotene (**Supplementary Tables 5, 6**). In all varieties, an increase of β,β-xanthophylls and phytoene occurred during ripening concomitantly with a decrease of lutein and α- and β-carotene. The main difference in flavedo between the red and the standard varieties was the concentration of phytoene, which was between 2- and 20-times higher in K and R (**Supplementary Tables 5, 6**). On the other hand, the content of violaxanthin in December and January in the flavedo of K and R was between 30% and 50% lower than in the corresponding standard oranges, respectively.

### Plastid ultrastructure in the juice vesicles of Navel and Kirkwood oranges

**Figure 4:**
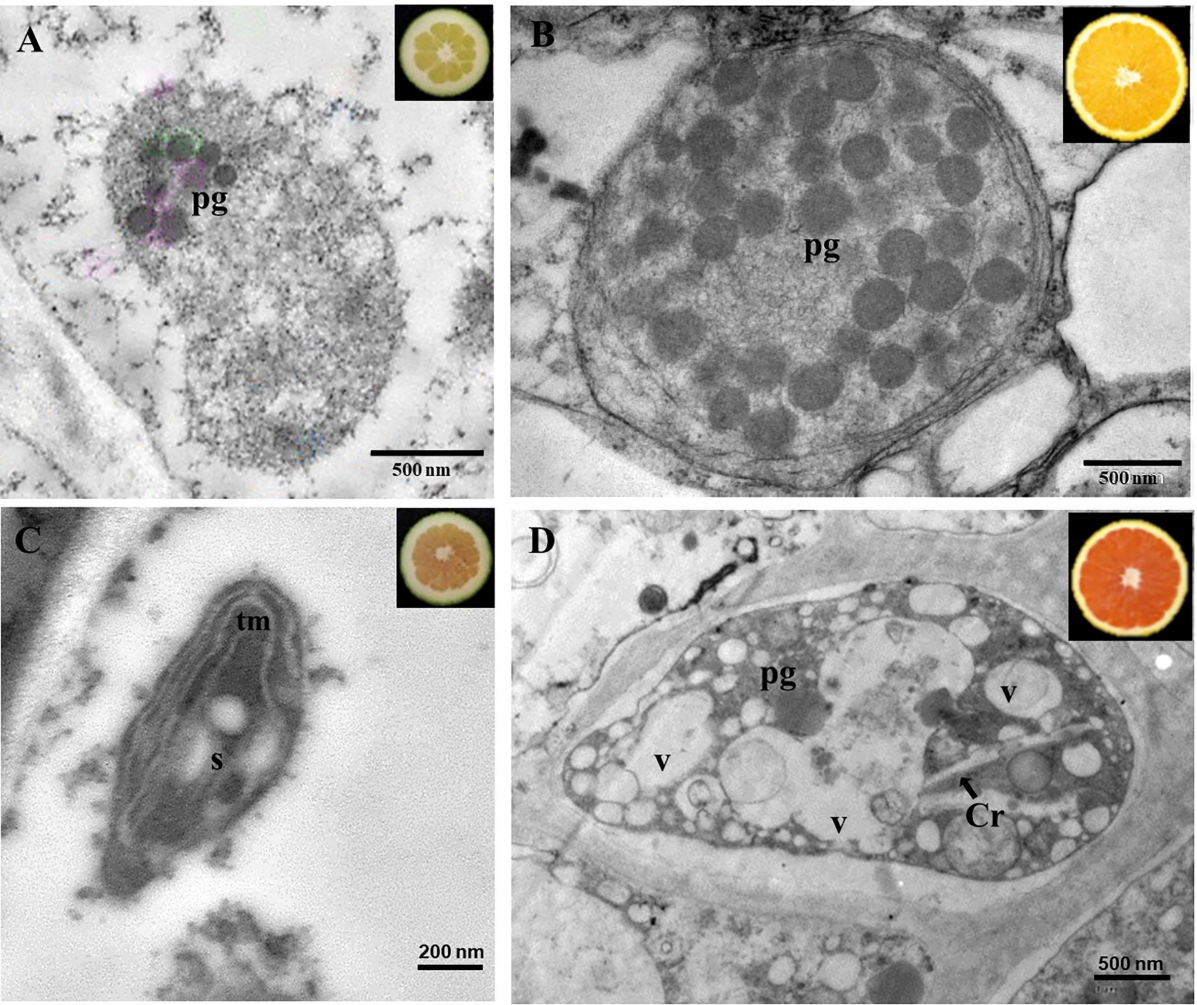
Transmission electron microscopy pictures of chromoplasts of the pulp of immature **(A)** and mature **(B)** fruits of Navel, and immature **(C)** and mature **(D)** fruits of Kirkwood. Pg, plastoglobuli; tm, tubular membranes; V; vesicles; S, starch; Cr, crystals of lycopene.

To obtain a more thorough insight into the alteration in carotenoid complement of mutant fruits, the plastids ultrastructure of juice vesicles cells from the N and K immature (**Figures 4A, C** **)** and mature (**Figures 4B, D** **)** fruits were examined by transmission electron microscopy (TEM). Differences in the ultrastructure of plastids were identified between both genotypes and ripening stages (**Figures 4A–D**). Plastids of immature fruits of standard N oranges presented a globular-type structure with plastoglobuli (**Figure 4A**), while chromoplasts of mature fruits were characterized by the increase in the number and size of plastoglobuli (**Figure 4B**). However, plastids of immature K fruits showed the presence of internal tubular membranes, starch granules and a lower number or absence of plastoglobuli (**Figure 4C**). In addition, juice vesicles of mature mutant fruits contained chromoplasts with different morphology and organization than those of standard oranges: reduced number of plastoglobuli, long structures compatible with lycopene crystals, and a high number of round electron-lucent vesicles (**Figure 4D**). Thus, the changes in carotenoid content and composition in the red-fleshed orange mutants are also associated with alterations in plastid ultrastructure.

### Expression of genes involved in carotenoid metabolism in standard Navel and Valencia, and the red-fleshed Kirkwood and Ruby oranges during fruit development and maturation

**Figure 5:**
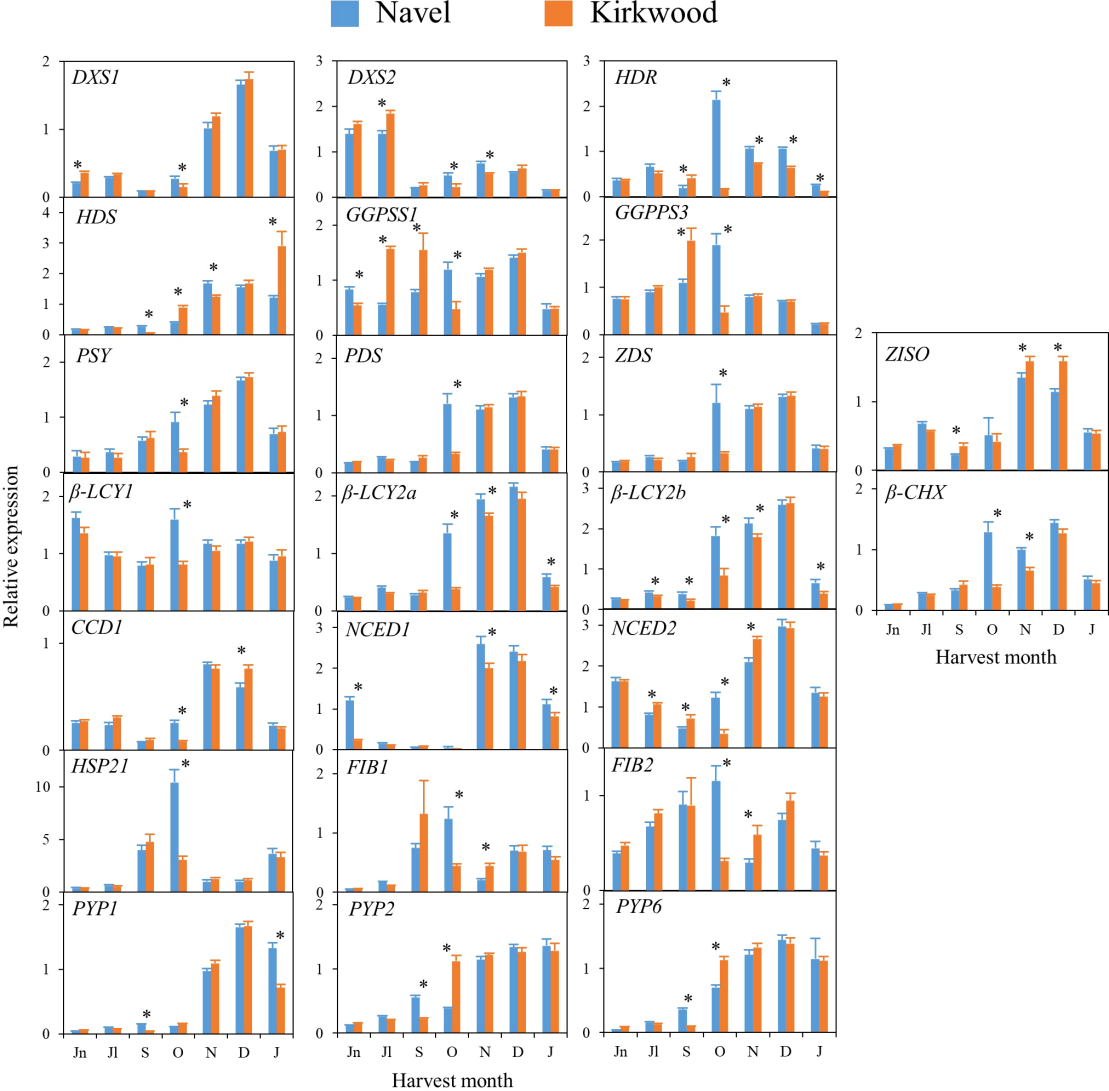
Relative expression levels of genes of carotenoids precursors (*DXS1*, *DXS2*, *HDR*, *HDS* and *GGPPS1* and *GGPPS3*), carotenes and xanthophylls biosynthesis (*PSY, PDS, Z-ISO, ZDS, β-LCY1*, *β-LCY2a*, *β-LCY2b*, *β-CHX*), carotenoids catabolism (*CCD1b*), ABA biosynthesis (*NCED1* and *NCED2*), carotenoid-associated protein (*HSP21*, *FIB1* and *FIB2*) and xanthophyll esterification (*PYP1, 2* and *6*) in the pulp of Navel and Kirkwood during fruit development and maturation. Jn, June; Jl, July; S, September; O, October; N, November; D, December; J, January. Data are the mean of three biological replicates ± SD. Asterisks indicate significant differences between Navel and Kirkwood varieties by unpaired Student’s t-test (p < 0.05).

**Figure 6:**
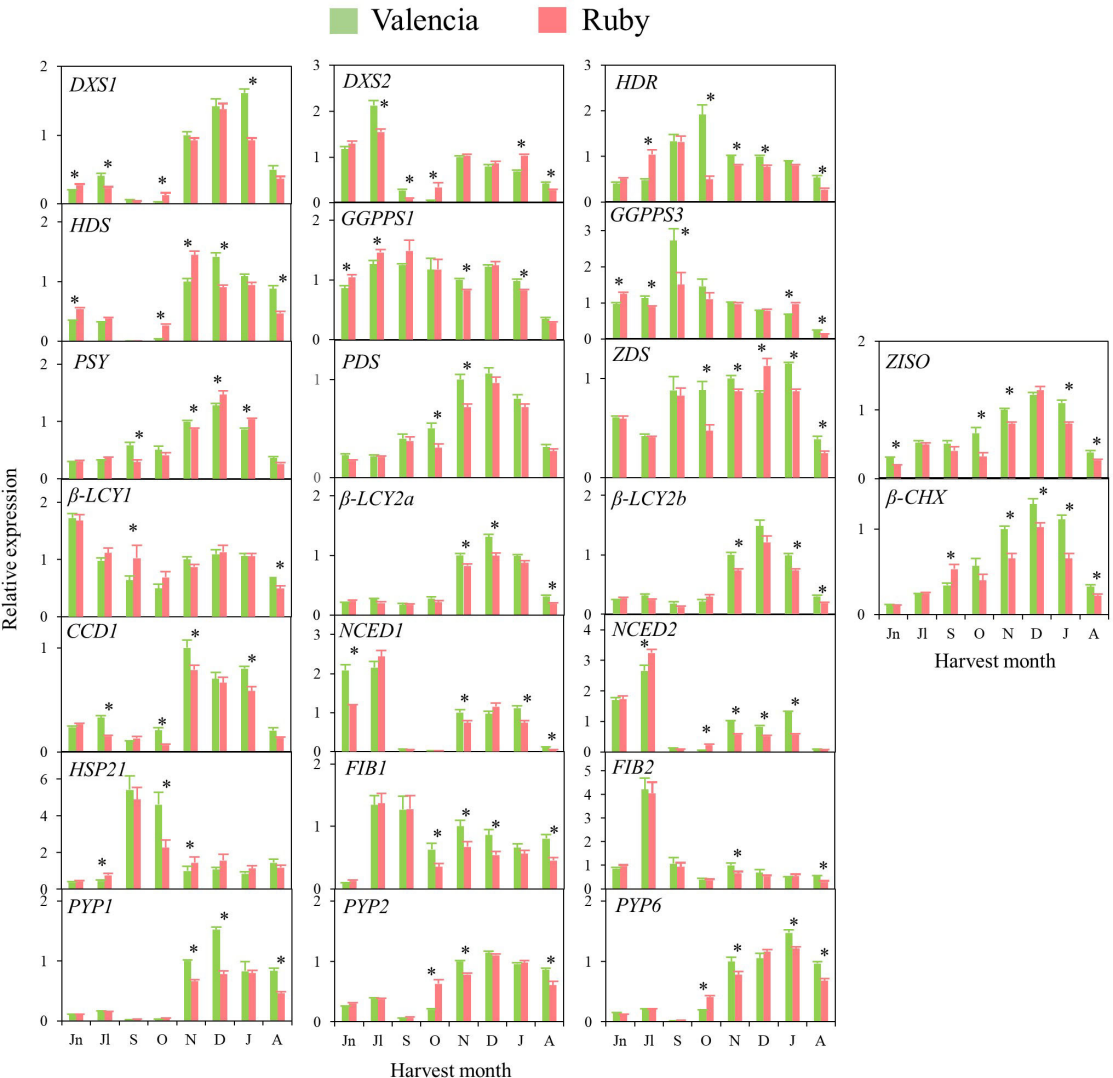
Relative expression levels of genes of carotenoids precursors (*DXS1*, *DXS2*, *HDR*, *HDS* and *GGPPS1* and *GGPPS3*), carotenes and xanthophylls biosynthesis (*PSY*, *PDS*, *Z-ISO*, *ZDS*, *β-LCY1*, *β-LCY2a*, *β-LCY2b*, *β-CHX*), carotenoids catabolism (*CCD1b*), ABA biosynthesis (*NCED1* and *NCED2*), carotenoid-associated protein (*HSP21*, *FIB1* and *FIB2*) and xanthophyll esterification (*PYP1, 2* and *6*) in the pulp of Valencia and Ruby during fruit development and maturation. Jn, June; Jl, July; S, September; O, October; N, November; D, December; J, January; A, April. Data are the mean of three biological replicates ± SD. Asterisks indicate significant differences between Valencia and Ruby varieties by unpaired Student’s t-test (p < 0.05).

The transcriptional profiling of 26 genes related to carotenoid metabolism and accumulation was analysed in the pulp and flavedo of the mutants K and R, and the corresponding standard varieties N and V during fruit development and maturation (**Figures 5**, **6** and **Supplementary Figures 2, 3** **)**. Most of the genes showed a similar expression pattern between the standard oranges and their respective red-fleshed varieties, even though some variety- or ripening-specific differences were observed (**Figures 5**, **6**). Transcripts of *β-CHX2* were not detected in any of the samples analysed, and *PSY2*, *ϵ-LCY* and *CCD4b* were only detected in flavedo.

In general, in the four varieties similar time–course gene expression patterns during development and ripening were found. The genes of the carotenoid precursors *DXS1, HDR* and *HDS* experienced a transient up-regulation in the pulp during fruit development or maturation, while expression of *DXS2, GGPS1* and *GGPS3* was sustained or decreased (**Figures 5**, **6**). Notably, the expression of *PSY, PDS, Z-ISO, ZDS*, *β-LCY2 (a* and *b* alleles*)* and *β-CHX*, enzymes involved in the serial conversion of phytoene to β,β-xanthophylls, were up-regulated during ripening. The maximum level of expression for these genes was reached approximately in November and January, depending on the orange group (Navel or Valencia) (**Figures 5**, **6**), coinciding with the maximum increment in the accumulation of β,β-xanthophylls. Transcript levels of *β-LCY1* did not substantially change during ripening. The pulp of both mutants showed significantly higher expression of *DXS1* than the standard varieties in June (early stage of fruit development), however, these differences were not sustained during ripening. The expression of other carotenoid biosynthetic genes in the pulp of K and R revealed a delay in their up-regulation compared to the standard varieties (**Figures 5**, **6**). In October, K fruits showed lower relative expression of *HDR*, *GGPS1*, *GGPS3*, *PSY*, *PDS*, *ZDS*, *β-LCY1*, *β-LCY2* than N fruits, but in November only *β-LCY1, β-LCY2* and *β-CHX* genes were reduced in K compared to N (**Figure 5**). Similarly, the expression of *PDS, ZDS, Z-ISO*, *β-LCY1*, *β-LCY2* and *β-CHX* genes was also lower in R compared to V in October and November (**Figure 6**). For most of the carotenoid biosynthetic genes, transcript levels decreased at later stages of ripening (**Figures 5**, **6**).

Regarding the genes encoding for 9-*Z*-epoxycarotenoid dioxygenases (*NCED1* and *2*), which are involved in the catabolism of 9-*Z*-xanthophylls and the production of ABA, a relative high expression was detected at immature stages (June-July) that decreased progressively until the beginning of maturation, to increase again between November and January (**Figures 5**, **6**). Interestingly, in June, both K and R showed significantly lower expression of *NCED1* than N and V oranges, respectively. Some differences were observed in the expression of *NCED1* and *2* genes at other ripening stages between the red and the blond oranges, but without a consistent trend. Additionally, *CCD1*, which also encodes for a carotenoid cleavage dioxygenase enzyme, showed a transient up-regulation with a maximum expression in November or December. To elucidate whether the massive accumulation of carotenoids in the red-fleshed mutants may be associated with alterations in proteins related to carotenoid storage, genes involved in carotenoid-sequestering structures *(FIBs)*, chaperone activity (*HSP21)* and esterification of xanthophylls *(PYPs)* were examined. The expression profiles of *FIB* genes did not follow a definite trend, but in general, their levels of expression were down-regulated along maturation. The expression of *HSP21* experienced a sudden increase concomitantly with the shift of external colour from green to yellow (October), showing a major induction in N than in K. This work examined for the first time the expression of *PYP1, 2* and *6* genes, the putative enzymes responsible for xanthophylls fatty acid esterification during fruit ripening, in the red-fleshed oranges. In general, *PYPs* genes exhibited an up-regulation pattern during ripening but higher levels of *PYP2* and *PYP6* transcripts were detected in both red-fleshed varieties in October. Nevertheless, minor differences in the expression of *PYPs* were observed between varieties at later stages of ripening (**Figures 5**, **6**).

The expression of 26 genes related to carotenoid metabolism and accumulation was also analysed in flavedo of the four orange genotypes. As a general observation, in the four varieties the relative transcript levels for all the genes was between 2 and 50-times higher in the flavedo than in the pulp. The transcripts of *PSY2*, *ϵ-LCY* and *CCD4b* were only detected in flavedo. Similarly to the expression profiling described for the pulp, most of the genes evaluated in the flavedo displayed a comparable expression pattern in the standard than in the red-fleshed varieties. However, some differences were identified for certain genes and ripening stages: genes of carotenoids precursors *DXS1*, *DXS2* and *GGPS3* were down-regulated during ripening. In contrast, *HDR*, *HDS* and *GGPS1*, and most of the carotenoid biosynthetic genes (*PSY, PDS, ZDS, Z-ISO*, *β-LCY2a*/*b* and *β-CHX*) were progressively up-regulated during maturation (**Supplementary Figures 2, 3**). The expression levels of *ϵ-LCY* and *β-LCY1* genes were maintained relatively constant during maturation. In Navel oranges, the genes involved in carotenoid catabolism, *NCEDs*, *CCD1* and *CCD4b*, were up-regulated, whereas in Valencia oranges displayed a fluctuating pattern. Finally, *FIBs*, *HSP21* and *PYPs* genes were up-regulated in the flavedo of all varieties and, interestingly, their expression was substantially higher in the flavedo of mature fruits of K than N (**Supplementary Figure 2**).

### Sequences of *ϵ-LCY*, *β-LCY1* and *β-LCY2* in standard Navel and Valencia, and the red-fleshed Kirkwood and Ruby oranges

The large amounts of carotenes upstream lycopene in the pulp of K and R fruits compared with the standard N and V envisage alterations in the cyclization of lycopene from the very early stages of fruit development. Alterations in the sequences of *ϵ-LCY*, *β-LCY1* and/or *β-LCY2* leading to non-functional proteins could be, indeed, a primary cause for lycopene accumulation in the red-fleshed oranges. To explore this hypothesis, the full-length coding sequences of *ϵ-LCY*, *β-LCY1* and/or *β-LCY2* of the red-fleshed varieties were examined for possible mutations. For each gene, two alleles, *a* and *b*, were identified: *β-LCY1*a (acc. OP441052), *β-LCY1*b (acc. OP441053), *β-LCY2a (*acc. OP441054*), β-LCY2b* (acc. OP441055*)*, *ϵ-LCYa* (acc. OP441056) and *ϵ-LCYb* (acc. OP441057) (**Supplementary Figure 4** and **Supplementary Table 7**), which agrees with the heterozygosity of the sweet orange genome (Wu et al., 2014). The alleles *a* and *b* of the *ϵ-LCY*, *β-LCY1*, *β-LCY2* genes differed in 5, 18 and 27 SNPs that resulting in 5, 10 and 21 amino acid changes, respectively (**Supplementary Table 7**). The sequence analyses of alleles *a* and *b* for each gene and variety revealed no differences between the standard and the red-fleshed genotypes.

### ABA and its catabolites in standard Navel and Valencia, and the red-fleshed Kirkwood and Ruby oranges during fruit development and maturation

**Figure 7:**
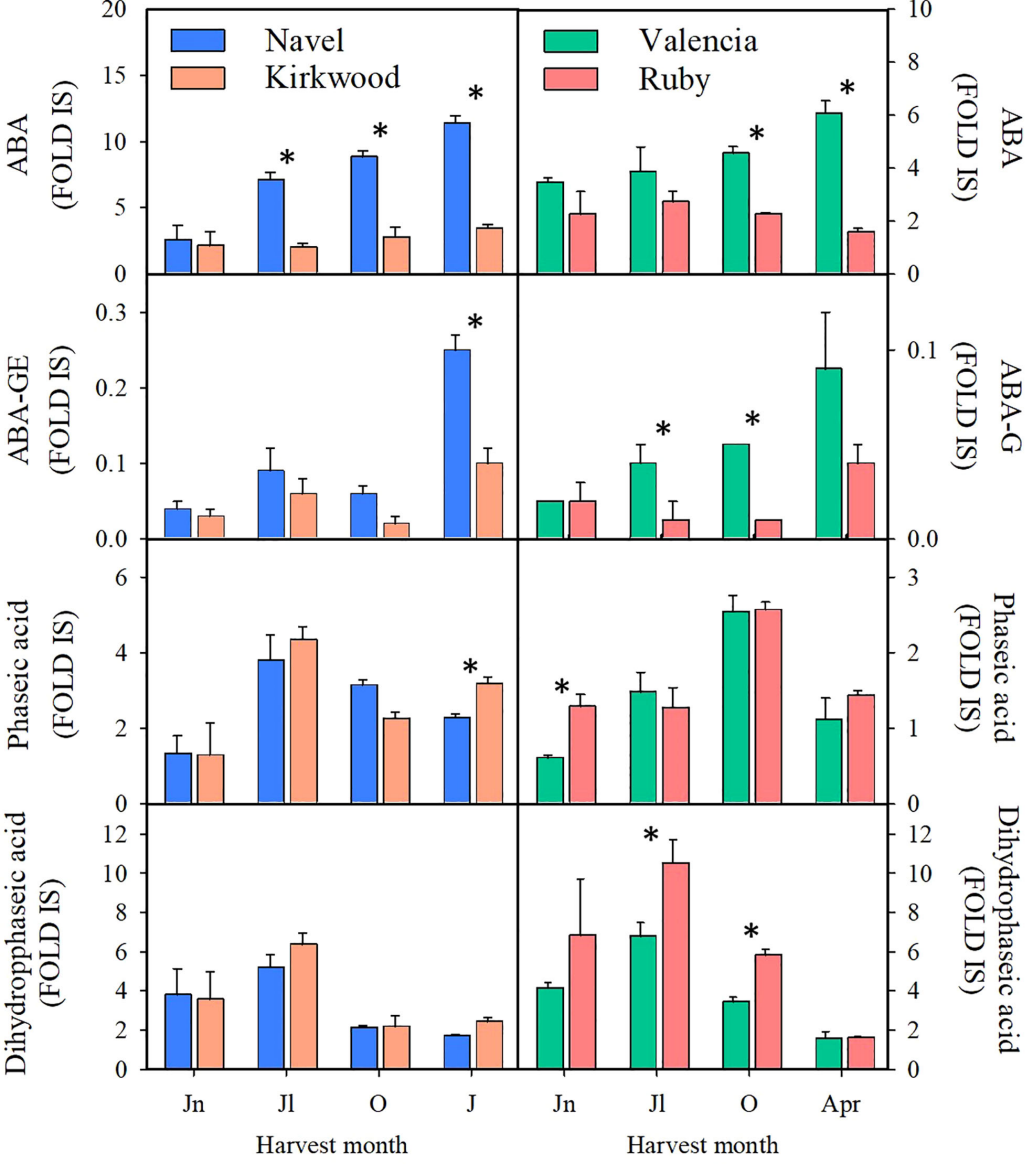
Content of abscisic acid (ABA), abscisic acid glucose ester (ABA-GE), phaseic acid and dihydrophaseic acid (Fold Internal Standard) in the pulp of Navel and Kirkwood (left panels) and Valencia and Ruby (right panels) during fruit development and maturation. Jn, June; Jl, July; O, October; J, January; Apr, April. Data are the mean of three biological replicates ± SD. Asterisks indicate significant differences between Kirkwood and Ruby and their respective standard varieties by unpaired Student’s t-test (p < 0.05).

The levels of abscisic acid (ABA), of its sugar-conjugated form ABA-glucosyl ester (ABA-GE), its catabolites, phaseic acid (PA) and dihydrophaseic acid (DPA), were determined in the pulp of the four orange genotypes at four stages of fruit development and ripening by LC-HRMS analysis (**Figure 7**). ABA and ABA-GE levels increased in the standard varieties throughout fruit maturation showing the maximum accumulation in mature fruits. However, in the pulp of K and R the relative levels of ABA and ABA-GE remained constant during ripening. Therefore, the relative levels of ABA and ABA-GE were between 2 and 3-times higher in the pulp of standard oranges compared to their respective mutants at colour break and mature stage. The levels of PA and DPA in the red-fleshed and standard oranges followed a similar pattern during ripening with minor and non-consistent differences detected between varieties or ripening stages (**Figure 7**).

In flavedo, minor differences were detected between the red-fleshed and standard varieties. K contained lower levels of ABA, ABA-GE and DA than N fruits in October, while R and V fruits did not show significant differences in ABA or its catabolites (**Supplementary Tables 8, 9**).

### Chlorophylls and carotenoids composition in leaves of Navel and Valencia and the red-fleshed mutants Kirkwood and Ruby

Chlorophyll and carotenoid composition analyses in leaves revealed noticeable differences between the red-fleshed and standard orange varieties. (**Table 3**). More specifically, in the leaves of both mutants, the content of chlorophyll ‘a’ and ‘b’ were higher than in the standard varieties, resulting in total chlorophylls being 20-30% higher. Leaves of K and R presented larger total carotenoid concentrations than the corresponding N and V. Total carotenoids in K and R leaves accounted 317.19 µg g^-1^ FW and 306.67 µg g^-1^ FW, respectively, compared with 251.84 µg g^-1^ FW and 232.45 µg g^-1^ FW of N and V, respectively. Lutein was the main carotenoid in the leaves of all varieties, followed by violaxanthin, other β,β-xanthophylls, and α-carotene and β-carotene. K and R leaves contained elevated concentrations of α-carotene and lutein although not statistically significant for lutein in K and for α-carotene in R. Nevertheless, the most remarkable feature regarding carotenoid composition in leaves of both mutants was the large accumulation of phytoene, with an 8- and 6-fold increment with respect to N and V, respectively (**Table 3**).

**Table 3:** Chlorophylls and carotenoids content and composition (µg g^-1^ FW) in leaves of Navel, Kirkwood Navel, Valencia and Ruby varieties.

Chlorophylls (µg g^-1^ FW)	Navel	Kirkwood	Valencia	Ruby
Chlorophyll a	1116.65 ± 15.38*	1557.93 ± 60.74	1447.22 ± 7.00*	1773.95 ± 220.28
Chlorophyll b	413.29 ± 1.65*	426.03 ± 5.19	456.83 ± 1.33*	593.70 ± 1.65
Total chlorophylls	1529.95 ± 17.03*	1983.97 ± 65.93	1904.05 ± 5.66*	2367.66 ± 17.03
**Carotenoids (µg g^-1^ FW)**				
Phytoene	3.43 ± 0.28*	25.84 ± 4.97	3.09 ± 0.57*	17.59 ± 0.59
Lutein	140.19 ± 8.23	151.60 ± 8.51	124.27 ± 12.23*	164.44 ± 14.30
α-carotene	23.09 ± 3.78*	38.10 ± 5.31	19.12 ± 2.88	23.03 ± 2.82
β-carotene	17.27 ± 3.76	21.56 ± 4.72	23.18 ± 6.65	19.68 ± 2.36
Violaxanthin	46.63 ± 3.76	56.67 ± 3.61	36.29 ± 2.24	42.99 ± 3.56
Other β,β-xanthophylls	21.23 ± 2.53	23.42 ± 1.90	26.50 ± 0.40*	39.52 ± 2.83
Total carotenoids	251.84 ± 21.78*	317.19 ± 29.09	232.45 ± 5.91*	306.67 ± 30.25

The amount of violaxanthin represents the sum of all-*E*-violaxanthin, 9-*Z*-violaxanthin isomers. Other β,β-xanthophylls are the sum of antheraxanthin, zeaxanthin and neoxanthin. Data are the mean of three biological replicates ± SD. Asterisks indicate significant differences between Kirkwood and Ruby and their respective standard varieties by unpaired Student’s t-test (p<0.05).

### Carotenoid composition in stems of Valencia and the red-fleshed mutant Ruby

**Figure 8:**
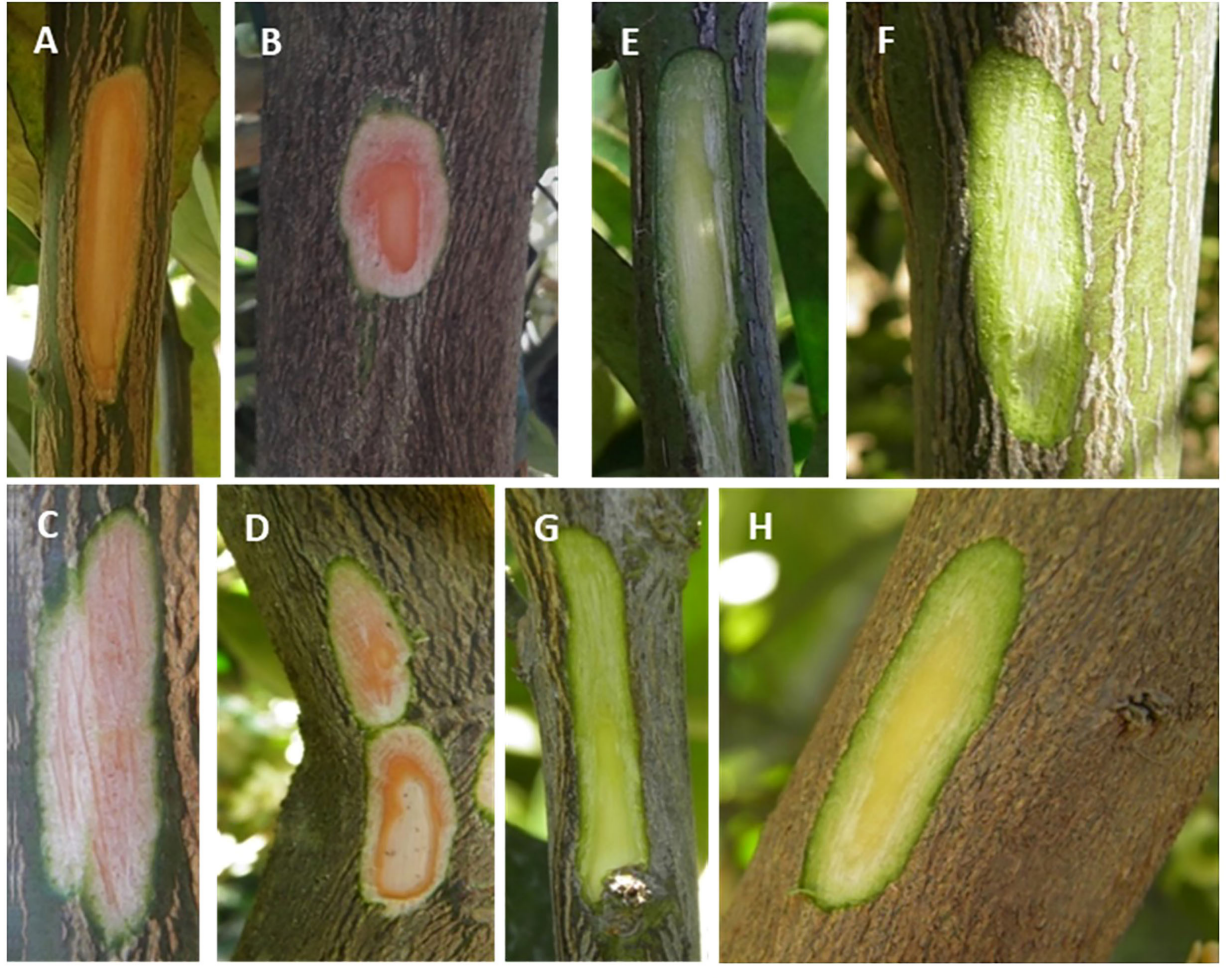
Representative pictures of young stems of Kirkwood **(A**, **B)**, Ruby **(C**, **D)** Navel **(E**, **F)** and Valencia **(G**, **H)** orange trees grown in South Africa. A small thin layer of the external bark was longitudinally removed to see the colouration of the inner bark and wood.

A remarkable field observation that was made in the case of the stems of the Ruby and Kirkwood trees under South African growing conditions regarded an unusually reddish colouration at the inner side of the bark, specifically the layers of the phloem/cambium, between the bark and xylem/wood tissue (a trait that was not observed under Spanish growing conditions) (**Figures 8A–D**). This distinct coloration could be easily distinguished by peeling the bark from the branches, which was more noticeable in older shoots and branches. The coloration continued in the whole tree until the graft union, whereafter the normal pale colour associated with citrus xylem was visible. By contrast, the same tissue in standard trees had an absence of any pigmentation and showed the characteristic green-yellow colouration of normal citrus branches (**Figures 8E-H**). Since this unusual red pigmentation of the inner layer of the stems of citrus trees has not been previously reported, we inferred that it might be related to the accumulation of lycopene in these mutants. Therefore, the carotenoid composition was analysed in bark and wood tissues of the standard Valencia orange and its red mutant R from trees growing in Citrusdal (South Africa) (**Table 4**). The bark (containing the phloem/cambium) and xylem (wood) tissues were separated and analysed independently (**Table 4**). In both genotypes, the concentration of carotenoids in the bark was higher than in the xylem tissue, and the differences between tissues were greater in the standard Valencia (74-times higher in bark) than in R (3.5-fold higher). The carotenoid content in bark and xylem of R was between 3 and 62 times higher than in the respective tissues of V. Moreover, both tissues of the R accumulated large concentrations of phytoene and significant amounts of phytofluene compared to V. Interestingly, low but still detectable amounts (<1 μg g^-1^ DW) of lycopene were quantified in the bark, while trace levels were detected in the xylem. As in the fruit pulp, a significantly lower concentration of xanthophylls was found in the bark of R compared to that of V. Interestingly, the bark and xylem of the red-fleshed variety also contained 20% less ABA and of DA and PA than those of the standard V (**Table 4**).

**Table 4:** Carotenoid content and composition (µg g^-1^ DW), and ABA, ABA-GE and ABA catabolites (Fold Internal Standard) levels in bark and wood of young stems of Valencia late (A) and Ruby Valencia mutant (B) orange trees.

	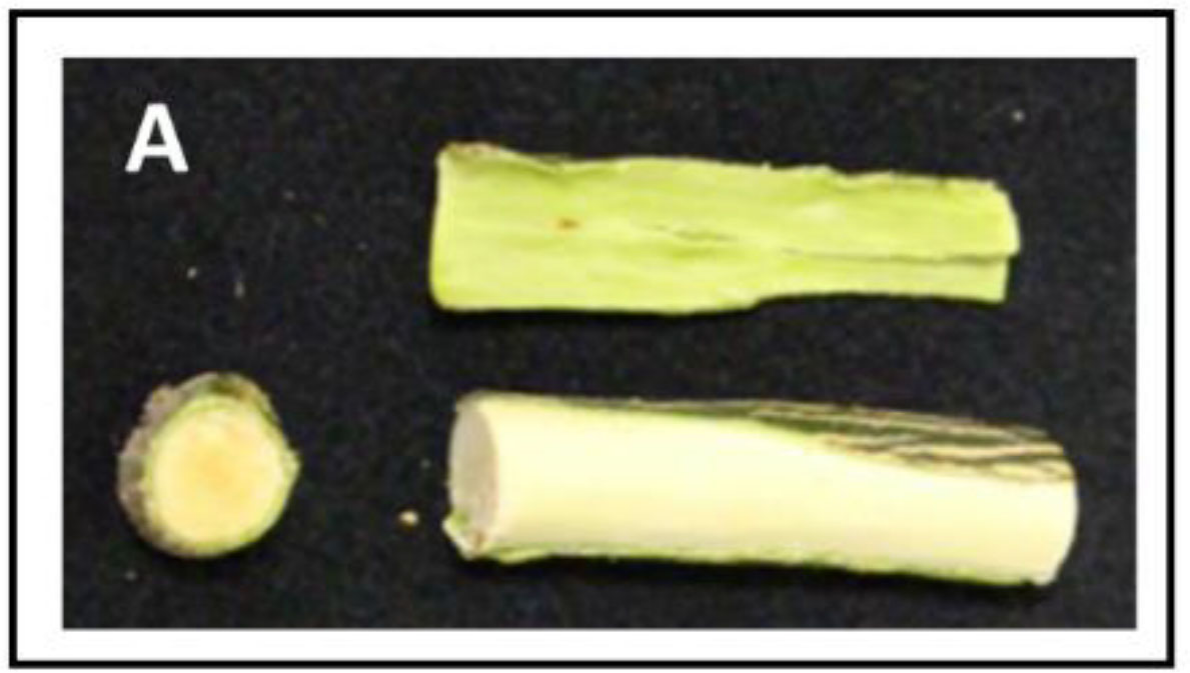		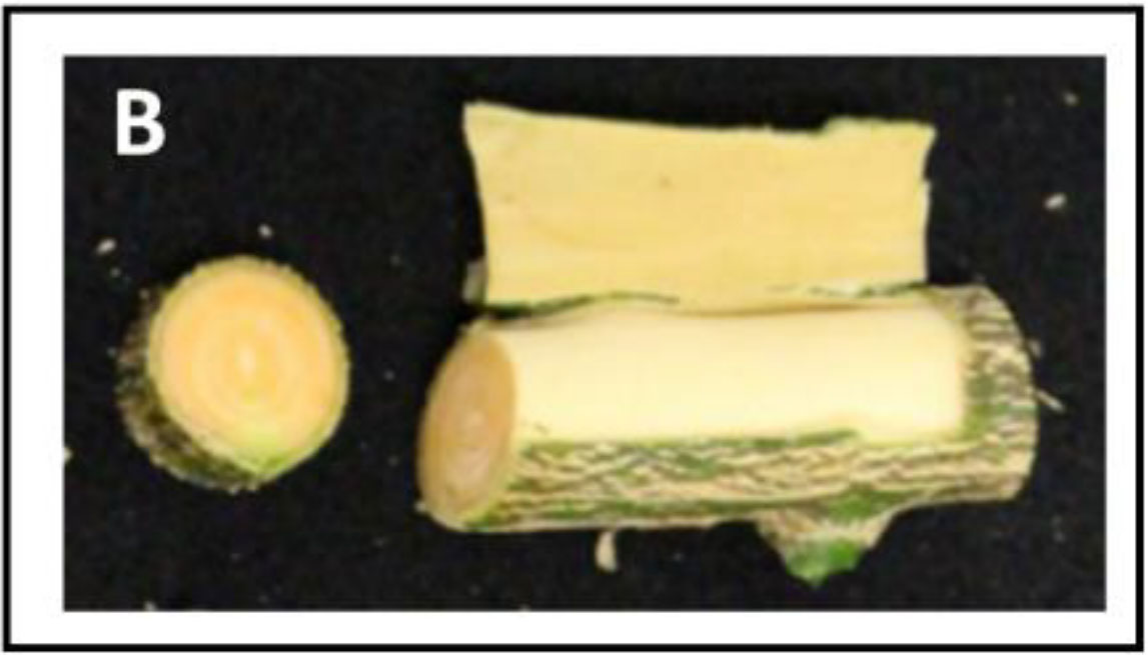	
Carotenoids (µg g^-1^ DW)	Valencia		Ruby	
	Bark	Wood	Bark	Wood
Phytoene	5.45 ± 1.81*	1.16 ± 0.05*	389.45 ± 20.58	139.01 ± 32.51
Phytofluene	nd	nd	38.35 ± 1.38	5.10 ± 3.31
α-carotene	2.01 ± 1.00	nd	4.27 ± 1.00	nd
Lutein	104.99 ± 4.14*	1.09 ± 0.11	56.57 ± 3.78	nd
β-carotene	4.93 ± 1.20	nd	4.30 ± 0.64	nd
Lycopene	nd	nd	0.65 ± 0.08	traces
Antheraxanthin	4.72 ± 2.67	nd	nd	nd
Violaxanthin	31.56 ± 1.56*	0.10 ± 0.10	11.46 ± 0.20	nd
Neoxanthin	23.16 ± 0.86*	nd	11.66 ± 3.81	nd
Total carotenoids	176.82 ± 8.63*	2.35 ± 0.04*	516.70 ± 28.29	146.29 ± 35.76
**ABA and ABA-related metabolites**(Fold IS)				
ABA	0.17 ± 0.04*	0.16 ± 0.01*	0.03 ± 0.01	0.03 ± 0.01
ABA-GE	nd	nd	0.04 ± 0.01	nd
DA	19.60 ± 1.54*	0.16 ± 0.02	4.83 ± 0.26	0.15 ± 0.01
PA	1.80 ± 0.14*	0.02 ± 0.01	0.16 ± 0.01	0.03 ± 0.01

The amount of violaxanthin corresponds to the sum of all-*E*-violaxanthin, all-*Z*-violaxanthin isomers and luteoxanthin. ABA, abscisic acid; ABA-GE, abscisic acid-glucose ester; DA, dihydrophaseic acid; PA, phaseic acid. Nd, no detected. Pictures represent the tissues used for carotenoid and ABA analyses. Data are the mean of three biological replicates ± SD. Asterisks indicate significant differences between Valencia and Ruby varieties by unpaired Student’s t-test (p<0.05).

This exceptional trait in branch and shoot tissue of the red-fleshed orange varieties led us to hypothesize that other lycopene-accumulating citrus species could also exhibit lycopene accumulation in these tissues. To explore this hypothesis, branches of three grapefruit (*Citrus paradisi*) varieties with different capacity to accumulate lycopene in the pulp and cultivated under similar environmental and nutritional conditions (IVIA, Valencia, Spain) were peeled off and examined. The Marsh grapefruit, which contains very low concentration of carotenoids in the pulp (Gross, 1987; Alquézar et al., 2013; Lado et al., 2015), did not show any distinctive colouration in the phloem/cambium. Nevertheless, Rio Red and Star Ruby, grapefruit varieties which accumulate lycopene in the pulp, exhibited a noteworthy reddish colouration in these tissues **(** **Supplementary Figure 5**). Altogether, these observations suggest that the metabolic alteration leading to lycopene accumulation affects not only the flesh of the fruit (oranges and grapefruit), but also other plant tissues (as xylem, calyx or vascular bundles).

## Discussion

Accumulating lycopene in the pulp of citrus fruits is an unusual feature highly attractive to producers and consumers. Carotenoid metabolism in red-fleshed fruits of several citrus species, including grapefruit, pummelo, lemon and sweet orange, has been largely studied but the specific molecular mechanisms leading to lycopene accumulation in the pulp of sweet oranges has not yet been elucidated (Tadeo et al., 2020). The availability of two novel red-fleshed sweet orange mutants belonging to different genetic backgrounds (K to the Navel group and R to the Valencia group) allows for a reliable characterization of the carotenoid metabolism, to identify the molecular basis of the alteration leading to this particular phenotype. These red-fleshed mutants K and R were originally identified in South Africa (**Figure 2**) and were grafted and propagated in trees located in experimental orchards in Spain, retaining their stability as to their distinctive phenotypic alterations. This allows us to perform, under identical agronomic and climatic Mediterranean conditions, a comparative biochemical and molecular study of the carotenogenesis with standard Navel and Valencia genotypes during fruit development and maturation (**Figure 4**).

The red colouration of the pulp is the most remarkable phenotypic characteristic of K and R oranges. Other parameters analysed related to fruit development and maturation, as shape, external colour or internal maturity index (°Brix or acidity) were very similar to that of their corresponding standard varieties, indicating that the colour of the flesh is the major phenotypic alteration in these fruits (**Figure 3**; **Supplementary Tables 1, 2**).

Comparison of the carotenoid content and composition in both red-fleshed mutants revealed a similar exceptional profiling with respect to the standard varieties. In the pulp of K and R oranges, indeed, more than 80% of total carotenoids correspond to linear carotenes, with large amounts of phytoene and phytofluene, and moderate content of lycopene, which provides their characteristic red pigmentation (**Figures 2**, **3**). This unique carotenoid profile of mature K and V oranges resembles that previously reported for the red-fleshed orange Cara Cara (Xu et al., 2006; Liu et al., 2007; Alquézar et al., 2008; Rodrigo et al., 2015; Lu et al., 2017; Liu et al., 2021). However, this altered carotenoid profile is different to that of the red Hong Anliu mutant, derived from the Chinese Anliu sweet orange, which accumulates lycopene in the pulp without increasing other upstream carotenes (Liu et al., 2007; Xu et al., 2009), suggesting that the basis of Hong Anliu alteration is different to that occurring in other red orange genotypes.

The early reddish-pink shade and accumulation of carotenes (including lycopene) in the pulp of K and R does not seem to be a ripening-related event. The mutation is manifested from the initial stages of carotenogenesis and occurs much earlier than the upsurge of carotenoids during maturation (**Tables 1**, **2**). This early pigmentation in the pulp of the mutants has also been observed in Cara Cara (Alquézar et al., 2008; Lu et al., 2017), pink lemon (Lana et al., 2020) and some red pummelos (Yan et al., 2018; Promkaew et al., 2020). However, in other red-fleshed genotypes, as Hong Anliu orange (Liu et al., 2007), red grapefruits (Alquézar et al., 2013) and Red Guanxi pummelo (Liu et al., 2016), lycopene accumulates during fruit ripening but not at the early stages of development. It is worth noting that in the pulp of grapefruits (*C. paradisi*), pummelos (*C. maxima/grandis*) and lemons (*C. limon*), the carotenogenesis is different to that of orange-coloured varieties (oranges and mandarins), and these fruits accumulate lower carotenoid content with a virtual absence of β,β-xanthophylls (Tadeo et al., 2020). Thus, the differential pattern of carotenes accumulation suggests that the alteration in carotenoid metabolism leading to lycopene accumulation and the red pulp may differ among citrus species and varieties.

The carotenoid profiling between the standard and the red-fleshed oranges revealed that, besides the presence of lycopene in the pulp, a remarkable feature is the extremely high concentration of phytoene, reaching values of 80-125 μg g^-1^ FW in both flavedo and pulp tissues of mature fruits (**Tables 1**, **2**; **Supplementary Tables 5, 6**). The phytoene concentrations in the red-fleshed varieties are among the highest ever-reported in *Citrus* (Lado et al., 2015; Rodrigo et al., 2019) and other fruit and vegetable (Meléndez-Martínez et al., 2015). Phytoene and phytofluene are highly bioavailable and may be involved in health-promoting effects such as skin protection, antioxidants and cancer prevention (Engelmann et al., 2011; Mapelli-Brahm and Meléndez-Martínez, 2021). Regarding human consumption, the uptake of a single K or R orange would provide between 10-15 mg of phytoene, which is superior to the daily intake for most of the major carotenoids detected in humans (Mapelli-Brahm and Meléndez-Martínez, 2021). Therefore, these red-fleshed oranges are a very valuable source of colourless carotenes, in addition to lycopene, for human consumption and to develop novel food/feed as well as cosmetics, enriched in these carotenes.

Other interesting feature of the K and R carotenoid profile was the accumulation of ζ-carotene and neurosporene in the pulp (**Table 1**). These carotenes are intermediate products of the desaturation reactions from phytofluene to lycopene (**Figure 1**) and are not usually detected in the pulp of citrus (Rodrigo et al., 2013b). The accumulation of ζ-carotene and neurosporene has been reported in other citrus mutants with a partial blockage at initial steps of the carotenoid biosynthetic pathway (Alquézar et al., 2009; Alquézar et al., 2013; Rodrigo et al., 2019). In the context of these results, the accumulation of δ-carotene in the red-fleshed oranges is also of particular significance. The accumulation of this intermediate product in the synthesis of α-carotene (**Figure 1**), with only a ϵ-ring at one end, suggests a partial impairment in the *β*-ring cyclization in the pulp of these genotypes.

The pulp of the red mutants during maturation accumulated 2 to 3-times less violaxanthin than the standard varieties (**Tables 1**, **2**). This xanthophyll is the main end-product of the carotenoid biosynthetic pathway in sweet oranges (**Figure 1**) (Kato et al., 2004; Rodrigo et al., 2004; Tadeo et al., 2020). However, there is not a clear consensus on whether the accumulation of lycopene in red-fleshed mutants is associated with a reduced concentration of β,β-xanthophylls, since conflicting results have been reported in Hong Anliu and Cara Cara (Liu et al., 2007; Alquézar et al., 2008; Lado et al., 2015; Lu et al., 2017; Liu et al., 2021). The reduction of violaxanthin in the pulp of K and R oranges was accompanied by significant less ABA and its glycosylated form ABA-GE (**Figure 7**). A decrease in the ABA content in the pulp of red compared to standard varieties has been previously explained by a lower expression of *NCED1* and *NCED2* genes, the main enzyme regulating synthesis of ABA (Xu et al., 2009; Pan et al., 2012; Alquézar et al., 2013; Liu et al., 2021). However, it is likely that in the K and R mutants the reduced content of 9-Z-violaxanthin, the ABA precursor, may be limiting for the synthesis of this hormone rather than or in addition to, the expression of *NCEDs* (**Figures 5**, **6**). It has been recently suggested that the pulp of citrus fruit is an active site of ABA synthesis, where the hormone is rapidly metabolized towards the conjugated product ABA-GE (Liu et al., 2021). Our results in the pulp of K and R showed that the reduction in ABA-GE levels paralleled the decrease in ABA during ripening (**Figure 7**), in line with those of Cara Cara and Hong Anliu oranges (Liu et al., 2021). In summary, the reduction in ABA and its conjugated ABA-GE is a distinctive characteristic in red-fleshed citrus, suggesting a partial blockage in the carotenoid biosynthesis pathway limiting the flow to downstream metabolites. This limitation in ABA and ABA-GE synthesis appears not to be deleterious for fruit development and ripening, as most of the physiological processes analysed in the pulp of these fruit were not substantially altered in respect to the standard oranges.

The atypical accumulation of carotenoids in the K and V mutants was associated with altered morphology of the chromoplasts ultrastructure, which contained tubular membranes and lycopene crystalloid-like structures not observed in standard sweet oranges and an unusual distribution of plastoglobuli (**Figure 4**). Other studies have reported similar differences in the ultrastructure of chromoplasts from red-fleshed orange and grapefruit during fruit maturation (Lado et al., 2015; Lu et al., 2017). These changes in plastidial structures might not be the reason for lycopene accumulation and could more be likely the consequence of the anomalous carotenoid accumulation, which requires an alternative sub-cellular organization and organelle structure (Nogueira et al., 2013; Zeng et al., 2015; Lu et al., 2017). Furthermore, the chromoplasts of the pulp of K mature fruits showed the presence of round translucent vesicles (**Figure 4**) resembling those previously described in Pinalate orange mutant, likely containing large amounts of colourless carotenes (Lado et al., 2015). It is then possible that these vesicles may serve as storage structures for phytoene/phytofluene in the red-fleshed mutants

The analysis of flavedo and leaves of the red-fleshed varieties revealed that the alterations in the carotenoid composition of these tissues are different to the pulp and may provide clues about the molecular basis of this trait. The carotenoid biosynthesis in flavedo of citrus fruit is regulated differently and independently compared to the pulp (up to 10-times higher in mature oranges) (**Supplementary Table 5, 6**) (Tadeo et al., 2020). Carotenoid composition in the flavedo and leaves of other red-fleshed oranges has been scarcely studied (Liu et al., 2007; Alquézar et al., 2008), and in this study we found that, unlike the pulp, the levels of violaxanthin, ABA and ABA-GE in the flavedo of mature K and R fruits were similar to those of the standard varieties (**Supplementary Tables 5–9**). Interestingly, the leaves of these varieties, as in the flavedo, presented an elevated content of phytoene (**Table 3**). These results indicate that the carotenoid profile is also altered in flavedo and leaves of K and R genotypes, but not to such extend as in the pulp.

The carotenoid profile and the high content of linear carotenes in the pulp of the K and R mutants resembled those found in Cara Cara fruits (Alquézar et al., 2008; Lu et al., 2017; De Ancos et al., 2020; Liu et al., 2021). In Cara Cara, it has been suggested that these alterations may be related to increased levels of carotenoid precursors or to an enhanced PSY activity (Alquézar et al., 2008). Nevertheless, subsequent studies using inhibitors of PDS (NFZ) and lycopene cyclase (CPTA) demonstrated that PSY activity is not higher in Cara Cara compared to standard Navel oranges (Lu et al., 2017). The transcriptional analysis of the carotenoid precursors genes in K and R oranges showed arbitrary differences that generally were not consistently maintained during the whole development and ripening (**Figures 5**, **6**). Therefore, a transcriptional enhancement of the MEP pathway genes appears not to be the primary basis for the carotenoid alteration in K and R oranges. Some studies proposed that low transcript level of *β-LCY2* gene or mutations in amino acids critical for the *β-LCY2* activity as the molecular mechanisms of lycopene accumulation in Red Anliu orange (Lu et al., 2016) and red grapefruits (Alquézar et al., 2009; Alquézar et al., 2013). Similarly, a reduction in *LCYs* gene expression was proposed in pink lemon and red pummelos (Lana et al., 2020; Promkaew et al., 2020; Tatmala et al., 2020). Nonetheless, neither key differences in the expression of *β-LCYs* nor mutations in their sequences were associated with lycopene accumulation in Cara Cara (Alquézar et al., 2008; Lu et al., 2017). In other plants, different mechanistic alterations have been proposed to be responsible for lycopene accumulation, i.e., mutations in the *β-LCY2* gene that originate reduced enzyme activity or, alternatively, post-translational modifications of β-LCY enzymes could be responsible for a deficient cyclization of lycopene (Blas et al., 2010; Devitt et al., 2010). In red watermelon varieties, the levels of β-LCY protein correlated negatively with lycopene accumulation (Zhang et al., 2020). The expression of the β-*LCY2a/b* genes was significantly lower in the pulp of K and R during maturation, which may favour the accumulation of upstream carotenes, including lycopene. However, transcript levels of *β-LCY2a/b*, and also of *β-LCY1* and *ϵ-LCY*, do not show consistent differences between the red and the standard oranges at the early stages of fruit development to substantiate the profound alterations in the carotenoid composition already observed at these physiological stages (**Figures 5**, **6**; **Supplementary Figures 2, 3**). Moreover, we also explored potential mutations in the *ϵ-LCY*, *β-LCY1* and *β-LCY2* gene sequence that may jeopardize the cyclization of lycopene. As expected, due to the heterozygosity of sweet oranges, two alleles were identified for each *LCY* gene. Nevertheless, there was no difference in the sequence for any *LCY* gene between the red-fleshed and the blond oranges (**Supplementary Figure 4** **)**. Therefore, it seems unlikely that alterations in the transcriptional profiling of *LCYs* genes or mutations in their coding sequences are responsible for the unusual carotenoid accumulation in the K and R oranges. It would be plausible to suggest the existence of other mechanisms, still unknown, associated with an accurate lycopene cyclase activity or post-transcriptional modifications of *LCY* genes. Further investigations would be necessary to confirm these hypotheses.

The transcriptional analysis of other carotenoid biosynthetic genes in the red-fleshed and standard oranges only showed differences in the expression of a few genes, which did not explain the differences in the carotenoid content and composition (**Figures 5**, **6**; **Supplementary Figures 2, 3**). Lu et al. (2017) suggested that potential alterations in the carotenoid degradation capacity could explain the increase in carotenoids content in Cara Cara fruits. To explore this possibility, we analysed the expression of several carotenoid cleavage dioxygenases genes (*CCD1*, *CCD4b* and *NCEDs*) involved in the catabolism of carotenoids (**Figures 5**, **6**; **Supplementary Figures 2, 3**). Our data indicate that the red-fleshed genotypes do not show regular differences in transcript levels of these genes. Furthermore, the lower accumulation of xanthophylls and ABA detected in the pulp of K and R are not consistent with impaired carotenoid catabolism.

Carotenoid synthesis in fruit occurs concomitantly with the differentiation of chloroplasts into chromoplasts, leading to the development of sink structures to store newly produced carotenoids (Sun et al., 2018). Recently, we found overexpression of *FIB1*, *FIB2* and *HSP21* in the pulp of the pink-fleshed lemon, suggesting an enhanced carotenoid accumulation and storage capacity (Lana et al., 2020). The expression levels in the pulp of K and V (**Figures 5**, **6**) do not support the involvement of fibrillins and the *HPS21* chaperone in the unusual accumulation of carotenes in the red-fleshed oranges. Another critical process for the massive accumulation of xanthophylls in chromoplastic tissues is their esterification with fatty acids (Rodrigo and Zacarias, 2019). The esterification of xanthophylls is a ripening-regulated event in citrus fruits that is tightly linked to the stability and accumulation of xanthophylls in chromoplastic tissues (Ma et al., 2017; Zacarías-García et al., 2021). To explore if this process might be altered in the red-fleshed mutants, we examined for the first time the expression of three *PYP*/*XAT* genes likely responsible for the esterification of xanthophylls in fruits (Zacarías-García et al., 2021) in K and R oranges. The expression of *PYP* genes in pulp and flavedo of the four genotypes assessed in this work showed an up-regulated pattern during ripening, and more importantly, no differences between the red and the blond oranges were observed. Taken together, we might conclude that the differential and usual accumulation of carotenoids in the red-fleshed K and V orange is not related to alterations in their capability for storage in specialized organelles or for esterification.

One of the most striking phenotypic features of the red-fleshed mutants is the reddish-pink colour of the calyx and its vascular axe, the vascular bundles of the albedo (**Figures 3B–G**), and the bark, particularly, the phloem layer of the shoots/branch (**Figure 8**). The woody tissue of the stem (comprising the cambium, the xylem and the pith) in the mutants also showed a pink/red shade, suggesting an unusual carotenoid accumulation in respect to standard orange stems. The bark of standard branches is an active carotenoid-accumulating site with high concentrations of lutein and lower levels of β,β-xanthophylls. However, despite the wood being almost devoid of carotenoids, the bark and wood accumulate similar amounts of ABA, indicating that the pathway is operative in both tissues, although the bark accumulates larger carotenoids (more than 80-times higher than the wood) (**Table 4**). Interestingly, the carotenoid content in the bark and wood of the mutant branches was remarkably high, mainly by increments in phytoene (70 and 120-times, respectively), phytofluene, and the presence of lycopene, which explains the reddish colouration of these tissues. By contrast, the metabolites downstream to lycopene were substantially reduced in the mutant, i.e., xanthophylls (2-3-fold reduction) and ABA and its catabolites (5-fold reduction). To our knowledge, this is the first time that the carotenoid content and composition, and ABA, its stored form (ABA-GE) and catabolites have been reported in *Citrus* stems (**Table 4**). Kuromori et al. (2014) demonstrated that phloem cells in vascular tissue of Arabidopsis are an active site of ABA synthesis that can be transported to other target cells. These observations revealed that the alteration in the carotenoid composition in these mutants is not restricted to fruit tissues, but manifests in other tissues undergoing active carotenoid and ABA synthesis. Considering that the bark of standard orange trees is very active for the synthesis of carotenoids and ABA, we hypothesize that a blockage or bottleneck in the lycopene cyclization step in the pathway would induce accumulation of upstream carotenes, lycopene and also a downstream reduction in ABA and its catabolites. On the other hand, the observation that the vascular bundles of the central axis of the fruit and the phloem of the branches in grapefruit similarly exhibited red colouration to their ability to accumulate lycopene in the pulp (**Supplementary Figure 5**), corroborates our hypothesis, suggesting that this may be a trait associated with the red-fleshed genotype, not exclusively in oranges, but also in other *Citrus* sp.

In summary, gene expression analysis of the carotenoid metabolism, including genes of carotenoid precursors, biosynthesis, catabolism and storage, during fruit development and ripening did not reveal conspicuous alterations in the K and R oranges that might explain their anomalous carotenoid composition in the pulp in comparison with the standard genotypes. Biochemical data of carotenoid composition in pulp and stems, particularly the increase of upstream carotenes (phytoene, phytofluene) and lycopene, as well as δ-carotene, and the reduced content of downstream products such as violaxanthin and ABA, firmly suggests impairment at the lycopene β-cyclization step in these mutants. The mutation is also manifested in the leaves and flavedo of the red-fleshed oranges, with the most evident trait being the higher levels of colourless carotenes. Therefore, the mutation is manifested in all the carotenogenic tissues/organs of the mutants, but with different consequences since this pathway is distinctly regulated in each tissue. In this context, it is important to highlight that in flavedo, the relative expression levels of virtually all carotenoid biosynthetic genes, including *β-LCYs*, is approximately 10-fold higher than in pulp. This fact results in an elevated capacity of this tissue to transform lycopene into β-carotene, which may partially alleviate the bottleneck and prevent lycopene accumulation in this tissue. Therefore, in other less carotenogenic tissues, i.e., pulp or stems, the limited cyclization of lycopene would have a more profound effect rendering extremely unbalanced carotenoids composition. Future studies, including the involvement of omics approaches, will be required to unravel the genetic basis of this intriguing reddish phenotype.

## Data availability statement

The datasets presented in this study can be found in online repositories. The names of the repository/repositories and accession number(s) can be found in the article/**Supplementary Material**.

## Author contributions

Conceptualization, PC, MR and LZ; methodology, JZ-G, MR and LZ; experimental work, formal analysis and data curation, JZ-G and GD, writing—original draft preparation, JZ-G; writing—review, JZ-G, PC, GD, MR, and LZ; supervision and funding acquisition, MR and LZ. All authors have read and agreed to the published version of the manuscript.

## Funding

This work was supported by the research grant RTI2018-095131-B-I00 funded by MCIN/AEI/10.13039/501100011033 (Spanish Government), “ERDF A way of making Europe” (Eu-ropean Union), EUROCAROTEN CA15136 (European COST_Action) and PROMETEO/2020/027 (Generalitat Valenciana, Spain). Jaime Zacarías-García is the recipient of a FDEGENT/2018/007 predoctoral scholarship from Generalitat Valenciana (Spain). The publication fee is partially supported by the CSIC Open Access Publication Support Initiative through its Unit of Information Resources for Research (URICI).

## Acknowledgments

The authors acknowledged ANECOOP S.COOP and Dr. Miguel Martínez for the facilities, support and the use of the experimental orchards and the provision of fruits. The authors also thank Biogold for allowing the use of the varieties for research purposes. To Citrus Rosso S.L. and Mr. Carlos Tornero for their support and contribution to this research throughout the years. The excellent technical assistance of Mª Carmen Gurrea and Inmaculada Carbonell is gratefully acknowledged.

## Conflict of interest

The authors declare that the research was conducted in the absence of any commercial or financial relationships that could be construed as a potential conflict of interest.

## Publisher’s note

All claims expressed in this article are solely those of the authors and do not necessarily represent those of their affiliated organizations, or those of the publisher, the editors and the reviewers. Any product that may be evaluated in this article, or claim that may be made by its manufacturer, is not guaranteed or endorsed by the publisher.
