# Supplementary material for: A comprehensive analysis of carotenoids metabolism in two red-fleshed mutants of Navel and Valencia sweet oranges (Citrus sinensis)
Source: Front Plant Sci. 2022 Oct 18;13:1034204. doi: 10.3389/fpls.2022.1034204 (PMC9623303; doi:10.3389/fpls.2022.1034204)
Supplement: Supplementary file 1 [file DataSheet_1.pdf]

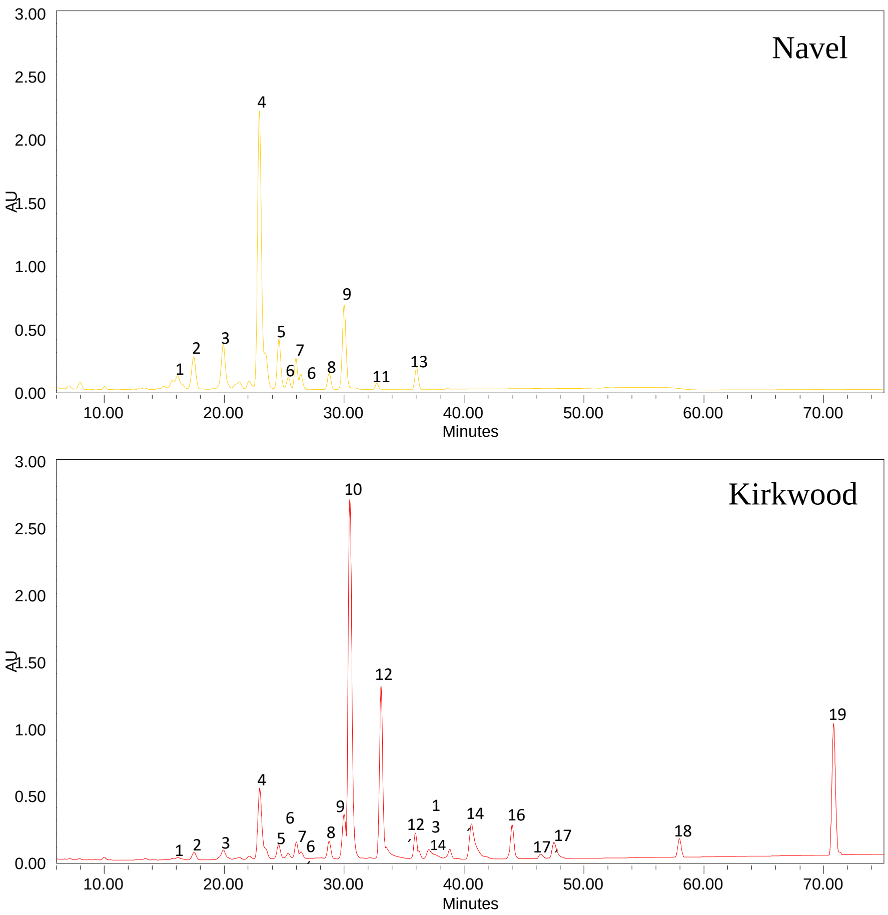

Supplementary Figure 1. HPLC profiles of saponified carotenoid extracts in pulp of fruit from (a) Washington Navel and (b) Kirkwood Navel Navel at full mature stage (January). All profiles are MaxPlot chromatograms (each carotenoid shown at its individual λ maxima). AU, Absorption units. The number peaks correspond to (1) 400-429-417; (2) All-*E*-violaxanthin; (3) Luteoxanthin; (4) 9-*Z*-violaxanthin; (5) 416-442-S; (6 and 6´) Mutatoxanthin isomers; (7) Lutein; (8) Zeaxanthin; (9) Antheraxanthin; (10) Phytoene; (11) α-Cryptoxanthin; (12) Phytofluene; (13) β-Cryptoxanthin; (14 and 14´) Neurosporene isomers (15) z-carotene; (16) β-carotene; (17 and 17´) d-carotene isomers; (18) 13-*Z*-lycopene; (19) all-*E*-Lycopene.

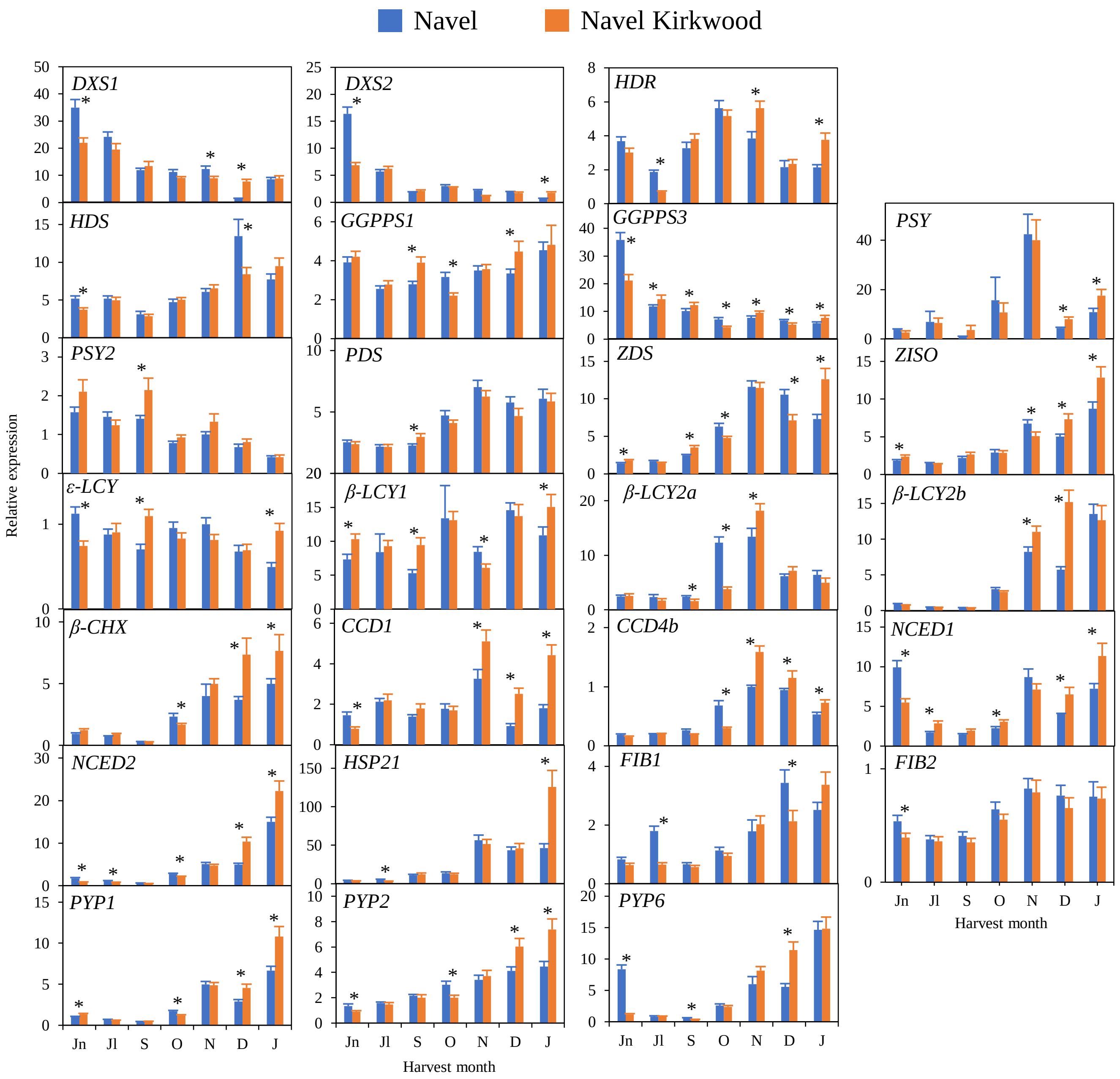

Supplementary Figure 2. Relative expression levels of genes of carotenoids precursors (*DXS1*, *DXS2*, *HDR*, *HDS* and *GGPPS1* and *GGPPS3*), carotenes and xanthophylls biosynthesis (*PSY*, *PSY2 PDS*, *ZISO*, *ZDS*, *β-LCY1*, *ε-LCY*, β-*LCY2a*, *β-LCY2b*, *β-CHX*), carotenoids catabolism (*CCD1* and *CCD4b*), ABA biosynthesis (*NCED1* and *NCED2*), carotenoid-associated proteins (*HSP21*, *FIB1* and *FIB2*) and xanthophyll esterification (*PYP1*, *2* and *6*) in the flavedo of Navel and Kirkwood during fruit development and maturation. Jn, June; Jl, July; S, September; O, October; N, November; D, December; J, January. Data are the mean of three biological replicates ± SD. Asterisks indicate significant differences between genotypes by unpaired Student's t-test ($p < 0.05$).

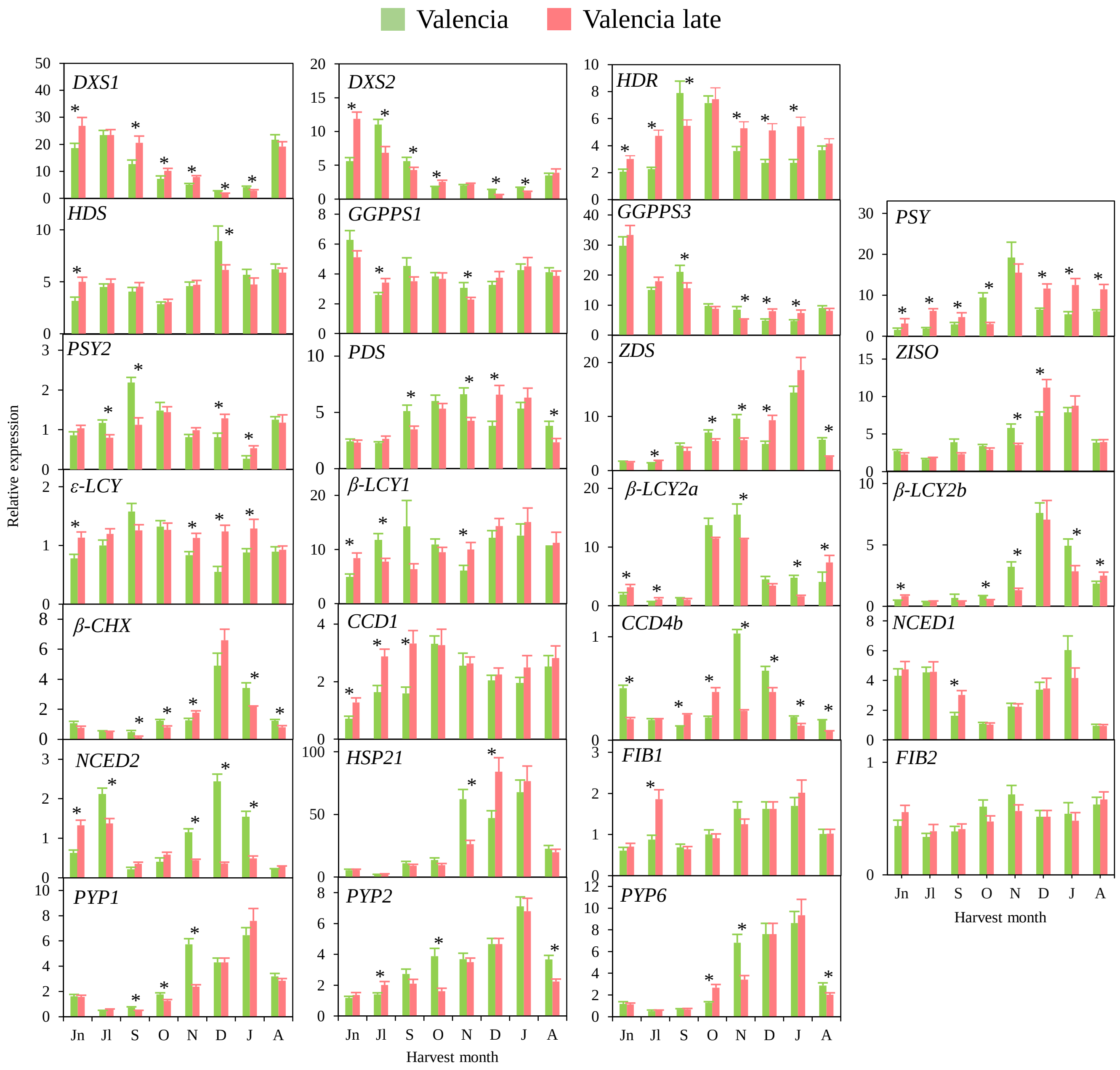

Supplementary Figure 3. Relative expression levels of genes of carotenoids precursors (*DXS1*, *DXS2*, *HDR*, *HDS* and *GGPPS1* and *GGPPS3*), carotenes and xanthophylls biosynthesis (*PSY*, *PSY2 PDS*, *ZISO*, *ZDS*, *β-LCY1*, *ε-LCY*, β-*LCY2a*, *β-LCY2b*, *β-CHX*), carotenoids catabolism (*CCD1* and *CCD4b*), ABA biosynthesis (*NCED1* and *NCED2*), carotenoid-associated proteins (*HSP21*, *FIB1* and *FIB2*) and xanthophyll esterification (*PYP1*, *2* and *6*) in the flavedo of Valencia and Ruby during fruit development and maturation. Jn, June; Jl, July; S, September; O, October; N, November; D, December; J, January; A, April. Data are the mean of three biological replicates ± SD. Asterisks indicate significant differences between genotypes by unpaired Student's t-test ($p < 0.05$).

Lycopene β-cyclase1a (β-LCY1a) (GenBank Acc OP441052)
ATGGATACTTTACTCAAAACTCATAACAAGCTTGAATTCTTGCCCCAAGTTCACGGGGCTTTGGAAAAAT
CCAGTAGTTTAAGCTCATTGAAGATTCAGAACCAGGAGCTTAGGTTTGGTCTCAAGAAGTCTCGTCAAAA
GAGGAATAGGAGTTGTTTCATTAAGGCTAGTAGTAGTGCTCTTTTGGAACTAGTTCCTGAAACCAAGAAG
GAAAATCTTGAATTTGAGCTTCCCATGTATGACCCATCAAAGGGCCTTGTTGTAGACCTAGCAGTTGTCG
GTGGCGGCCCGGCTGGGCTTGCTGTTGCTCAGCAAGTTTCAGGGGCGGGGCTTTCGGTTTGCTCGATTGA
TCCATCTCCCAAATTGATTTGGCCAAATAATTATGGTGTTTGGGTGGATGAATTTGAGGCCATGGATTTG
CTTGATTGCCTTGATACTACTTGGTCTGGTGCTGTTGTGCACATTGATGATAATACAAAGAAGGATCTTA
ATAGACCTTATGGGAGAGTTAATAGGAAGTTGCTGAAGTCGAAAATGCTGCAAAAATGCATAACCAATGG
TGTTAAGTTTCACCAAGCTAAAGTTATTAAGGTTATTCATGAAGAGTCCAAATCTTTGTTGATTTGCAAT
GATGGTGTGACAATTCAGGCAGCCGTGGTTCTTGATGCTACGGGGTTCTCTAGGTGTCTTGTCCAGTATG
ATAAGCCCTATAATCCAGGTTACCAAGTGGCATATGGAATACTAGCTGAGGTAGAACAGCACCCGTTTGA
TTTAGACAAGATGGTTTTCATGGATTGGAGAGATTCGCATCTGAACAACAATTCGCAGCTCAAAGAGGCA
AATAGCAAAATTCCTACTTTTCTTTATGCCATGCCCTTTTCGTCAAACAGGATATTTCTTGAAGAGACTT
CGCTAGTGGCGCGGCCTGGAGTGCCAATGAAAGATATCCAGGAAAGAATGGTGGCTAGATTAAAGCACTT
AGGCATAAAAGTTAAAAGCATTGAAGAGGATGAGCATTGTGTCATTCCGATGGGTGGGCCCCTTCCAGTG
CTTCCTCAAAGAGTTGTTGGAATAGGTGGTACCGCTGGGATGGTGCACCCTTCAACTGGCTATATGGTGG
CAAGGACTTTAGCTGCGGCTCCTATTGTTGCAAATGCAATCGTTCGAAGCCTCAGTTCTGACAGAAGCAT
TTCAGGACACAAATTGTCTGCTGAAGTTTGGAAAGATTTGTGGCCCATAGAAAGGAGAAGGCAAAGGGAG
TTCTTCTGTTTTGGTATGGATATCCTGCTCAAACTTGACTTACCTGCCACTAGAAGGTTTTTCGATGCTT
TTTTTGATCTGGAGCCTCGTTATTGGCATGGTTTCTTATCATCGAGATTGTTTCTCCCCGAGCTTTTAGT
TTTTGGGCTTTCTCTATTCTCACATGCCTCTAATACTTCTAGGCTAGAGATCATGGCAAAGGGCACTCTT
CCTTTGGTTAACATGATCAACAACTTGGTACAAGATACAGATTAA

Lycopene β-cyclase1b (β-LCY1b) (GenBank Acc OP441053)
ATGGATACTGTACTCAAAACTCATAACAAGCTTGAATTCTTGCCCCAAGTTCACGGGGCTTTGGAAAAAT
CCAGTAGTTTAAGCTCATTGAAGATTCAGAACCAGGAGCTTAGGTTTGGTCTCAAGAAGTCTCGTCAAAA
GAGGAATATGAGTTGTTTCATTAAGGCTAGTAGTAGTGCTCTTTTGGAGCTAGTTCCTGAAACCAAGAAG
GAAAATCTTGAATTTGAGCTTCCCATGTATGACCCATCAAAGGGCCTTGTTGTAGACCTAGCAGTTGTCG
GTGGTGGCCCAGCTGGGCTTGCTGTTGCTCAGCAAGTTTCAGAGGCGGGGCTTTCGGTTTGCTCGATTGA
TCCATCTCCCAAATTGATTTGGCCAAATAATTATGGTGTTTGGGTGGATGAATTTGAGGCCATGGATTTG
CTTGATTGCCTTGATACTACTTGGTCTGGTGCTGTTGTGCACATTGATGATAATACAAAGAAGGATCTTG
ATAGACCTTATGGCAGAGTTAATAGGAAGTTGCTGAAGTCGAAAATGCTGCAAAAATGCATAACCAATGG
TGTTAAGTTCCACCAAGCTAAAGTTATTAAGGTTATTCATGAAGAGTCCAAATCTTTGTTGATTTGCAAT
GATGGTGTGACAATTCAGGCTGCCGTGGTTCTTGATGCTACGGGATTCTCTAGGTGTCTTGTGCAGTATG
ATAATCCCTATAATCCAGGTTACCAAGTGGCATATGGAATACTAGCTGAGGTAGAAGAGCACCCGTTTGA
TTTAGACAAGATGGTTTTCATGGATTGGAGAGATTCGCATCTGAACAACAATTCGGAGCTCAAAGAGGCA
AATAGCAAAATTCCTACTTTTCTTTATGCCATGCCCTTTTCGTCAAACAGGATATTTCTTGAAGAGACTT
CGCTAGTGGCGCGGCCTGGAGTGCCAATGAAAGATATCCAGGAAAGAATGGTGGCTAGATTAAAGCACTT
AGGCATAAAAGTTAGAAGCATTGAAGAGGATGAGCATTGTGTCATTCCGATGGGTGGGCCCCTTCCAGTG
CTTCCTCAAAGAGTTGTTGGAATAGGTGGTACCGCTGGGATGGTGCACCCTTCAACTGGCTATATGGTGG
CAAGGACTTTAGCTGCGGCTCCTATTGTTGCAAATGCAATCGTTCGAAGCCTCAGTTCTGACAGAAGCAT
TTCAGGACACAAATTGTCTGCTGAAGTTTGGAAAGATTTGTGGCCCATAGAAAGGAGAAGGCAAAGGGAG
TTCTTCTGTTTTGGTATGGATATCCTGCTCAAACTTGACTTACCTGCCACTAGATGGTTTTTCGATGCTT
TTTTTGATCTGGAGCCCCGTTATTGGCATGGTTTCTTATCATCGAGATTGTTTCTCCCCGAGCTTTTAGT
TTTTGGGCTTTCTCTATTCTCACATGCCTCTAATACTTCTAGGCTAGAGATCATGGCAAAGGGCACTCTT
CCTTTGGTTAACATGATCAACAACTTGGTACAAGATACAGATTAA

Lycopene β-cyclase2a (β-LCY2a) (GenBank Acc OP441054)
ATGGCAACTCTTCTTAGCCCGTTTTCTCCTTCTCCTTTAGCTAAAGTTTCGCAAATAATTGATTCAACAT
CATCACCTTCATTTTCCCTATTTCCATTAGGCCGCCAAAATGCATGTTCAAGAAAGGCGGATCATCATCA
TCATCACAGGATCCGGACAAGCAAGTTTGGTAACTTCCTAGAGTTGACACCGGAGTCGGTACCTGAATTC
TTAGACTTTGATCTCCCCTGGTTTCATCCGTCCGATCGTATTCGATATGACGTGATCATCATTGGCACTG
GACCAGCCGGCCTCCGTCTAGCTGAGCAAGTCTCATCGCGTCATAGTGTCAAGGTATGTTGTGTTGATCC

TTCACCTCTTTCTACGTGGCCTAACAACTATGGAGTTTGGGTTGATGAGTTTGAAGACATAGGACTTGTA
GACTGTTTGGACAAAACTTGGCCGATGACTTGTGTTTTTATTAATGATCACAAGACCAAGTATCTAGACA
GGCCCTACGGTCGTGTTAGTAGAAATATTTTGAAGACAAAGTTATTAGAGAATTGTGTTTCAAATGGAGT
TAAGTTTCATAAGGCTAAAGTTTGGCACGTGAATCATCAGGAGTTCGAGTCTTCGATTGTTTGTGATGAT
GGGAATGAGATTAAGGCTAGCTTGATTGTTGATGCTAGTGGCTTTGCTAGTAGTTTTGTTGAGTATGATA
AGCCAAGAAACCATGGATACCAAATTGCTCATGGGATTTTAGCTGAGGTTGAGAGTCACCCTTTTGATTT
AGACAAAATGGTTCTCATGGATTGGAGAGATTCCCATTTAGGGAATGAGCCTTACTTGCGAGCTAGCAAT
TTGAAGCTCCCAACTTTTCTCTATGCAATGCCATTTGATTCAAATTTGGTATTTTTAGAAGAAACATCTT
TGGTTAGTAGGCCAGTTTTGTCATATAAAGAGGTTAAGAGCAGAATGGCAGCGAGGTTAAGGCATATGGG
AATTAGAGTTAAAAGAGTGATTGAAGATGAAAAATGTTTGATTCCAATGGGAGGTCCTCTGCCTGTGATC
CCACAAAGTGTGATGGCTATTGGCGGCACGTCTGGTTTAATCCATCCTGCAACTGGGTATATGGTGGCTC
GAACCATGGCTCTGGCCCCTGCCTTGGCTGATGCAATAGCTGAATGCCTTGGCTCAACCAGGATGATCAG
AGGCAGGCCACTTCATCAGAAAGTGTGGAATGGGTTGTGGCCAATTGACAGAAGATGCAATAGGGAGTTT
TATTCATTTGGTATGGAGACTTTGTTGAAGCTGGATTTGAAGGGGACTAGGAGATTCTTTGATGCTTTCT
TTGATTTGAATCCTTACTACTGGCATGGGTTTCTGTCCTCAAGGTTGTCTCTTGCAGAGCTTGCTGGCCT
AAGCTTGTCTCTCTTTGGACACGCCTCGAATTCTTCCAGGTTGGATATTGTTACCAAGTGCCCTGTTCCT
CTGGTTAAAATGATGGGGAATCTTGCCCTTGAAACCATTTGA

Lycopene β-cyclase2b (β-LCY2b) (GenBank Acc OP441055)
ATGGCAACTCTTCTTAGCCCGTTTTCTCCTTCTCCTTTAGCTAAAGTTTCGCAAATAATTGATTCAACAT
CATCACATTCATTTTCGCTATTTCCATTAGGCCGCCAAAATGCATGTTCAAGAAAGGCGGATCATCATCA
TCATCACAGGATCCGGACAAGCAAGTTTGGTAACTTCCTAGAGTTGACACCGGAGTCGGAACCTGAATTC
TTAGTCTTTGATCTCCCCTGGTTTCATCCGTCCGATCGTATTCGATATGACGTGATCATCATTGGCACTG
GACCTGCCGGCCTCCGTCTAGCTGAGCAAGTCTCATCGCGTCATGGTATCAAGGTATGTTGTGTTGATCC
TTCACCTCTTTCTACGTGGCCTAACAACTATGGAGTTTGGGTTGATGAGTTTGAAGACATAGGACTTATA
GACTGTTTGGACAAAACTTGGCCGATGACTTGTGTTTTTATTAATGATCACAAGACCAAGTATCTAGACA
GGCCCTACGGTCGTGTTAGTAGAAATATTTTGAAGACAAAGTTATTAGAGAATTGTGTTTTAAATGGCGT
TAGGTTTCATAAGGCTAAAGTTTGGCATGTGAATCATCAGGAGTTCGAGTCTTCGATTGTTTGTGATGAT
GGAAATGAGATTAAGGCTAGCTTGATTGTTGATGCTAGTGGCTTTGCTAGTAGTTTTGTTGACTATGATA
AGCCAAGAAACCATGGATACCAAATTGCTCATGGGATTTTAGCTGAGGTTGAGAGTCACCCTTTTGATTT
AGATAAAATGGTTCTCATGGATTGGAGAGATTCCCATTTAGGGAATGAGCCTTACTTGCGAGCTAGCAAT
TTGAAGCTCCCAACTTTTCTCTATGCAATGCCATTTGATTCAAATTTGGTATTTTTAGAAGAAACATCTT
TGGTTAGTAGGCCAGTTTTGTCATATAAAGAGGTTAAGAGAAGAATGGCAGCGAGGTTAAGGCATATGGG
AATTAGAGTTAAAAGAGTGATTGAAGATGAAAAATGTTTGATTCCAATGGGAGGTCCTCTGCCTGTGATC
CCGCAAAGTGTGATGGCCATTGGCTGCACGTCTGGTTTAGTCCATCCTTCAACTGGGTATATGGTGGCTC
GGACCATGGCTCTGGCCCCTGCGTTGGCTGATGCAATAGCTGAGTGCCTTGGCTCAACCAGGATGATCAG
AGGCAGGCCACTTCATCAGAAAGTGTGGAATGGGTTGTGGCCAATTGACAGAAGATGCAATAGGGAGTTT
TATTCATTTGGTATGGAGACTTTGTTGAAGCTGGATTTGAAGGGGACTAGGAGATTCTTTGATGCTTTCT
TTGATTTGAATCCTCACTACTGGCATGGGTTTCTGTCCTCAAGGTTGTCTCTTGCAGAGCTTGCTGGCCT
AAGCTTGTCTCTCTTTGGACACGCCTCGAATTCTTCCAGGTTTGATATTGTTACCAAGTGCCCTCTTCCT
CTGGTTAAAATGATGGGGAATCTTGCCCTTGAAACCATTTGA

Lycopene ε-cyclase a (ε-LCYa) (GenBank Acc OP441056)
ATGGAATACTACTGTCTTGGAGCTCGAAATTTCGCAGCAATGGCGGTATCTCCTTTCCCAACAGGGCGGG
CGCGAAGGAAAGCACTGAGAGTGAAAACCAAACAGAGTGCTGTAGATTGTAATCATAGTTCCTATAAAGT
GACAGCTAGAGCTACTAGTAATAATGCTGGTAGTGAGAGTTGCGTAGCCGTCAAAGAAGAAGATTATATT
AAGGCCGGTGGCTCTCAGCTTGTTTTCGTACAAATGCAGCAAAACAAGTCAATGGATAAACAGTCTAAAT
TAGCCGATAAGCTACCGCCAATATCAATTGGTAATGGTATTTTGGATTTGGTGGTGATTGGTTGTGGCCC
AGCTGGTCTTGCTTTGGCTGCAGAATCAGCGAAGTTGGGATTAAATGTTGGACTTATTGGCCCGGATCTC
CCTTTCACAAACAATTATGGTGTGTGGGAAGATGAATTTAGAGATCTTGGACTTGAAGGGTGTATCGAAC
ATGTCTGGAGAGACACAGTTGTATATATTGATGAAGATGAACCCATCTTGATTGGTCGTGCTTATGGACG
AGTTAGTCGACATTTGCTTCATGAAGAATTATTAAGAAGGTGTGTCGAGTCAGGTGTTTCATATCTTAGC
TCAAAAGTGGAAAGCATTACGGAATCTACCAGTGGTCATCGTCTTGTAGCTTGTGAACATGATATGATTG

TCCCCTGCAGGCTTGCTACTGTTGCTTCTGGAGCAGCATCAGGGAAGCTATTGGAATATGAGGTGGGGGG
TCCCAAAGTTTCTGTCCAAACAGCTTATGGTGTGGAGGTTGAGGTGGAAAATAATCCATATGATCCAAGC
CTTATGGTTTTCATGGACTACAGAGACTGTACTAAGCAAGAAGTTCCATCTTTTGAATCTGACAATCCAA
CATTTCTTTATGTCATGCCCATGTCTTCAACAAGAGTTTTCTTTGAGGAAACTTGTTTGGCATCGAAAGA
TGGTTTACGTTTTGACATATTGAAGAAAAAGCTCATGGCAAGGTTAGAGAGATTGGGAATCCAGGTTTTG
AAAACTTATGAAGAGGAATGGTCATATATTCCAGTTGGTGGTTCCTTACCAAATACAGAACAAAGAAACC
TCGCATTTGGTGCTGCTGCTAGCATGGTGCATCCAGCCACTGGCTACTCAGTAGTCAGATCACTGTCAGA
GGCTCCAAACTATGCTTCTGCAATTGCATATATATTGAAACACGATCATTCCAGAGGTAGACTTACACAT
GAACAAAGTAATGAGAATATCTCAATGCAAGCTTGGAATACTCTCTGGCCACAGGAAAGGAAGCGCCAAA
GAGCTTTTTTCCTCTTTGGACTAGCACTCATTTTGCAACTGGATATTGAGGGCATCAGGACATTCTTTCG
CACTTTCTTCCGATTACCCAAGTGGATGTGGCACGGTTTCCTTGGTTCTAGTCTCTCATCAGCCGATCTC
ATTCTATTTGCCTTCTATATGTTTATTATAGCACCAAATGATCTGAGAAAGTGCCTTATCAGACATCTAG
TTTCAGATCCAACTGGAGCAACTATGGTAAGAACATACCTGACTTTATAG

Lycopene ε-cyclase b (ε-LCYb) (GenBank Acc OP441057)
ATGGAATACTACTGTCTTGGAGCTCGAAATTTCGCAGCAATGGCGGTATCTCCTTTCCCAACAGGGCGGA
CGCGAAGGAAAGCACTGAGAGTGAGAACCAAACAGAGTGCTGTAGATTGTAATCATAGTTCCTATAAAGT
GACAGCTAGAGCTACTAGTAGTAATGCTGGTAGTGAGAGTTGCGTAGCCGTCAAAGAAGAAGATTATATT
AAGGCCGGTGGCTCTCAGCTTGTTTTCGTACAAATGCAGCAAAACAAGTCAATGGATAAACAGTCTAAAT
TAGCCGATAAGCTACCGCCAATATCAATTGGTAATGGTATTTTGGATTTGGTGGTGATTGGTTGTGGCCC
AGCTGGTCTTGCTTTGGCTGCAGAATCAGCGAAGTTGGGATTAAATGTTGGACTTATTGGCCCGGATCTC
CCTTTCACAAACAATTATGGTGTGTGGGAAGATGAATTTAGAGATCTTGGACTTGAAGGGTGTATCGAAC
ATGTCTGGAGAGACACAGTTGTATATATTGATGAAGATGAACCCATCTTGATTGGTCGTGCTTATGGACG
AGTTAGTCGACATTTGCTTCATGAAGAATTATTAAGAAGGTGTGTCGAGTCAGGTGTTTCATATCTTAGC
TCAAAAGTGGAAAGCATTACGGAATCTACCAGTGGTCATCGTCTTGTAGCTTGTGAACATGATATGATTG
TCCCCTGCAGGCTTGCTACTGTTGCTTCTGGAGCAGCATCAGGGAAGCTATTGGAATATGAGGTGGGGGG
TCCCAAAGTTTCTGTCCAAACAGCTTATGGTGTGGAGGTTGAGGTGGAAAATAATCCATATGATCCAAGC
CTTATGGTTTTCATGGACTACAGAGACTGTACTAAGCAAGAAGTTCCATCTTTTGAATCTGACAATCCAA
CATTTCTTTATGTCATGCCCATGTCTTCAACAAGAGTTTTCTTTGAGGAAACTTGTTTGGCATCGAAAGA
TGGTTTACCTTTTGACATATTGAAGAAAAAGCTCATGGCAAGGTTAGAGAGATTGGGAATCCAGGTTTTG
AAAACTTATGAAGAGGAATGGTCATATATTCCAGTTGGTGGTTCCTTACCGAATACAGAACAAAGAAACC
TCGCATTTGGTGCTGCTGCTAGCATGGTGCATCCAGCCACTGGCTACTCAGTAGTCAGATCACTGTCAGA
GGCTCCAAACTATGCTTCTGCAATTGCATATATATTGAAACACGATCATTCCAGAGGTAGACTTACACAT
GAACAAAGTAATGAGAATATCTCAATGCAAGCTTGGAATACTCTCTGGCCACAGGAAAGGAAGCGCCAAA
GAGCTTTTTTCCTCTTTGGACTAGCACTCATTTTGCAACTGGATATTGAGGGCATCAGGACATTCTTTCG
CACTTTCTTCCGATTACCCAAGTGGATGTGGCACGGTTTCCTTGGTTCTAGTCTCTCATCAGCCGATCTC
ATTCTATTTGCCTTCTATATGTTTATTATAGCACCAAATGATCTGAGAAAGTGCCTTATCAGACATCTAG
TTTCAGATCCAACTGGAGCAACTATGGTAAGAACATACCTGACTTTATAG

Supplementary Figure 4. Sequences of lycopene β-cyclase1 (β-LCY1), lycopene β-cyclase2 (β-LCY2) and lycopene e-cyclase (ε-LCY) alleles in Kirkwood, Navel, Valencia Ruby and Valencia oranges. Note that no difference in the sequence of each allele was observed between the four orange varieties. Only the coding sequence is shown. The SNPs between alleles a and b for each gene are marked by yellow.

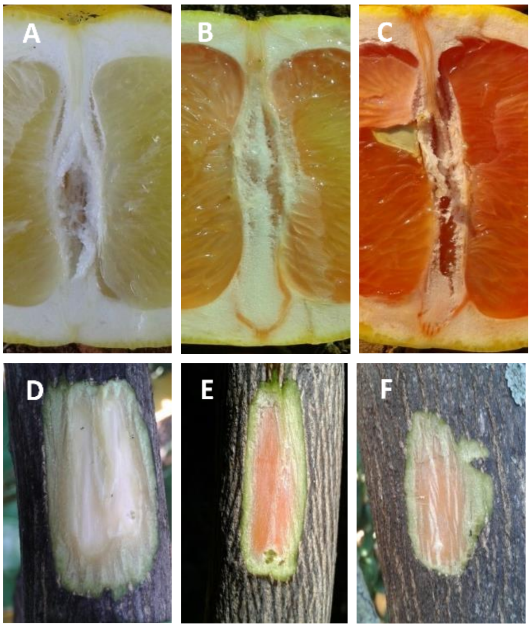

Supplementary Figure 5. Representative pictures of longitudinal sections of Marsh (A), Rio Red (B) and Star Ruby (C) grapefruits, and trunks of Marsh (D), Rio Red (E) and Star Ruby (F) grapefruit trees growing at the Citrus Germoplasm of the IVIA (Moncada, Valencia, Spain). Note the red colour of the notch and vascular axis of the fruit, and distinctive pigmentation of the inner bark and wood for each variety.
